# An association study of severity of intellectual disability with peripheral biomarkers of disabled children in a rehabilitation home, Kolkata, India

**DOI:** 10.1038/s41598-019-49728-3

**Published:** 2019-09-20

**Authors:** Aaveri Sengupta, Ujjal Das, Krishnendu Manna, Sushobhan Biswas, Siddhartha Datta, Amitava Khan, Tuhin Bhattacharya, Samrat Saha, Tapashi Mitra, Swapan Mukherjee, Anup K. Sadhu, Suhrita Paul, Saurabh Ghosh, Rakhi Dey Sharma, Sanjit Dey

**Affiliations:** 1 Department of Physiology, University of Calcutta, 92, A.P.C Road, Kolkata, 700009 West Bengal India; 2 Institute of Child Health, 11, Dr. Biresh Guha Street, Kolkata, 700017 West Bengal India; 3 EKO CT and MRI Scan Centre, Medical College and Hospitals campus, 88, College Street, Kolkata, 700073 West Bengal India; 4 Burdwan Medical College, Rajbati, Burdwan, 713104 West Bengal India; 5 Human Genetics Unit, Indian Statistical Institute, 203 B.T. Road, Kolkata, 700108 West Bengal India; 6 Department of Physiology, Belda College, Belda, Paschim Medinipur- 721424 West Bengal India

**Keywords:** Neurophysiology, Disability

## Abstract

The current investigation has identified the biomarkers associated with severity of disability and correlation among plethora of systemic, cellular and molecular parameters of intellectual disability (ID) in a rehabilitation home. The background of study lies with the recent clinical evidences which identified complications in ID. Various indicators from blood and peripheral system serve as potential surrogates for disability related changes in brain functions. ID subjects (Male, age 10 ± 5 yrs, N = 45) were classified as mild, moderate and severe according to the severity of disability using standard psychometric analysis. Clinical parameters including stress biomarkers, neurotransmitters, RBC morphology, expressions of inflammatory proteins and neurotrophic factor were estimated from PBMC, RBC and serum. The lipid peroxidation of PBMC and RBC membranes, levels of serum glutamate, serotonin, homocysteine, ROS, lactate and LDH-A expression increased significantly with severity of ID whereas changes in RBC membrane β-actin, serum BDNF, TNF-α and IL-6 was found non-significant. Structural abnormalities of RBC were more in severely disabled children compared to mildly affected ones. The oxidative stress remained a crucial factor with severity of disability. This is confirmed not only by RBC alterations but also with other cellular dysregulations. The present article extends unique insights of how severity of disability is correlated with various clinical, cellular and molecular markers of blood. This unique study primarily focuses on the strong predictors of severity of disability and their associations via brain-blood axis.

## Introduction

Intellectual disability (ID) refers to significantly sub-average intellectual functioning, resulting in concurrent impairments in adaptive behavior and manifests during the developmental period^1,2^. Recent clinical evidences have identified complications in ID to be reflected in blood cells. But the mechanistic link between disease severity and alterations in blood cell markers has not been untangled so far. ID has causal association with genetic or non-genetic factors such as, infection or intoxication during pregnancy, complications of delivery, or postnatal infection or trauma. Genetic causes are responsible for more than 50% of severely mentally disabled persons^3^. Intellectually disabled generally have reduced cognitive abilities and limited basic verbal abilities. The brain areas associated with social adaptation, motor and daily living skills are impaired more in generalized developmental disturbances^4^. Depending on the severity of the disease, they fail to decide or motivate themselves about their regular work and coping up with systemic stress.

All the deficits of abilities may arise from challenges against psycho-physiological and brain functions. However, the contributing factors and their role with the progression of disease severity are still under question. In particular, it is not explicit whether oxidative stress (OS) is a cause or a consequence behind severity of disability^5^. Recent reports implicated that ID is often related to deficits in glutathione antioxidant defense system in selective regions of the brain, which might be an influencing factor for OS, immune dysfunction, and apoptosis; particularly in the cerebral cortex and cerebellum^6^. Previous studies have suggested that OS and erythrocyte membrane alterations in ID children with autism are due to enhanced erythrocyte thiobarbituric acid reactive substances, urinary isoprostane, hexanoyl-lysine levels with a significant reduction of Na^+^/K^+^-ATPase activity, and a reduction of the erythrocyte membrane fluidity and alteration in the erythrocyte fatty acid membrane profile^7^. It is reported that hematological parameters like ferritin, iron, hemoglobin, hematocrit, and MCV are significantly lower in children with autistic disorder^8^. Also, Normal RBC morphology and expression of β-actin are altered in Rett syndrome (RTT) patients^9^. Thus, dysregulation in systemic redox homeostasis, damage in red blood cell integrity may contribute to severe disability.

It is reported that high levels of homocysteine (Hcy) and OS are generally linked with neuropsychiatric disorders. A study with autistic patients shows that there are higher levels of Hcy found in subjects with autism with respect to age-matched control group^10^. Another study reveals that plasma triglyceride and cholesterol are positively associated with the level of ID^11^.

Recent studies have shown that increased level of serum glutamate is found in male autistic subjects compared to their normal healthy counterparts. Furthermore, a positive correlation is found between serum glutamate levels and Autism Diagnostic Interview-Revised (ADI-R) social scores in autism patients^12^. More severe mental illness is selectively associated with GABA_A_ receptor-mediated inhibitory deficits^13^. In addition, there are numerous evidences that GABA plays an important role in the brain to combat with stress, the most important vulnerability factor in many mental disorders^14^. On the other hand, recent studies indicate that disability often correlates with hyperserotoninaemia^15^. Another report suggests that elevated blood serotonin is a logical marker in case of autism spectrum disorder and it plays significant role in multiple brain network as well as in neurodevelopment^16^.

A number of reports suggest a relationship between BDNF and the functioning of certain brain areas involved in attention and cognition. Some reports suggest that serum and cortical BDNF levels are positively correlated and that plasma BDNF levels directly reflect brain tissue levels^17^. Research reveals that serum BDNF level is nonspecifically reduced in acute severe mental illness (SMI) patients and increases during inpatient treatment^18^.

Accumulating evidence has been found towards association of dysregulation in central and peripheral immune system functioning with the severity of aberrant behavior and more impaired developmental and adaptive function^19^. Pro-inflammatory cytokines are increased in mentally disabled patients with epilepsy and in those subjects with enhanced neutrophil-lymphocyte ratio. Interleukin (IL)-6 is involved in physiological brain development and in many neurological disorders. Another finding has suggested that expressions of serum TNF-α and IL-6 and some other cytokines are increased in severe case of disability^20^. It is also reported that autistic patients show altered pattern of peripheral blood cytokines. Another literature indicates that depressed individuals have higher circulating neutrophils and lower lymphocytes compared to healthy group^21^. Thus, the neuroimmune molecules were shown to play roles in physiological development of brain as well as in several neurodevelopmental disorders.

Developmental and intellectual disability may predispose as crucial phenomenon for some physiological and cellular biomarkers. Consequently, the oxidative status in intellectually disabled individuals might be for derangement of systemic homeostasis, impairment in metabolic flux, cytoarchitectural alterations and to some extent immunomodulation. In our present study, we report for the first time, the identification and association of peripheral prognostic markers with increasing severity of neuro-developmental disorders.

## Results

### Classification of ID children according to IQ scores

Subjects (N = 45) were categorized in three groups (Mild, moderate and severe) based on their IQ scores using Stanford Binet Intelligence Scale and phenotypic severity (Table 1). IQ levels for mild were considered as 50–70, moderate 35–49 and severe 20–34.

**Table 1:** Basic information related to age (years), body weight (Kg), existing pathophysiology and behavioral aspects of male ID children (N = 45) of three respective groups categorized on the basis of their intellectual functioning and phenotypic severity.

Category	Age (years)	Gender	Body weight (Kg)	Pathophysiology	Behavioral Characteristics
Mild	13.3 ± 0.68	Male	36 ± 3.26	Sleep problems, speech Problem digestive issues, muscle weakness.	Autistic behaviour, aloofness, avoiding behaviour, attention seeking behaviour
Moderate	10.5 ± 1.06	Male	35.5 ± 2.81	Speech problem, seizures, sleep problems.	Autistic behaviour, Scholastic Deterioration, speech problem, lack of verbal communication, stubborn in nature.
Severe	9.2 ± 0.9	Male	37.5 ± 2.49	Sleep problems, lethargic, no sense of urination, muscle weakness, speech problem.	Mental backwardness, regressed speech, poor body balance, avoiding behaviour, attention deficit problems, self harming behaviour, restless, biting behaviour.

### Differences among physical age (PA), mental age (MA) and social age (SA) between ID groups

**Figure 1:**
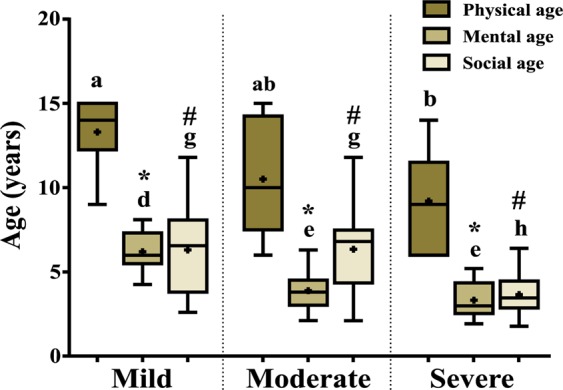
After categorization of the subjects are as mild (n = 15), moderate (n = 15) and severe (n = 15) on the basis of their IQ scores, their mental ages (MAs) were analyzed using Stanford Binet Intelligence Scale. Social ages (SAs) of subjects of three groups were evaluated by Vineland Social Maturity Scale (VSMS). Both the MAs and SAs were plotted against their chronological ages. The symbol asterisks (*) indicate significant difference in mental age compared to physical age either in mild, moderate or severe group. The symbol “#” represents significant difference in social age compared to physical age either in mild, moderate or severe group. The letters (a, b, c), (d, e, f) and (g, h, i) stand for significant differences in parametric values of physical age, mental age, social age of mild, moderate and severe group, respectively. The letters ‘ab’ indicate that the value is not significantly different compared to either ‘a’, or ‘b’.

MA and SA of disabled children were analyzed using Stanford Binet Intelligence Scale and Vineland Social Maturity Scale (VSMS) (Fig. 1). Significant differences found in PA vs. MA and PA vs. SA plots in case of severely affected group compared to other two groups. In case of severe intellectual disability, MA (2.75 ± 0.19) was lower than that of their PA (9.2 ± 0.9). SA has been found to be lower in severe group (6.34 ± 0.85) than PA (9.2 ± 0.9) in comparison to other groups.

### Changes in hematological parameters

Alterations in hematological parameters among the intellectually disabled categories were represented in Table 2. We observed that hemoglobin, HCT, MCV, neutrophil and lymphocyte counts exhibited significant difference between different groups of ID (see Supplementary Table S1). After performing multiple correction testing for the pair-wise comparisons, we found significant difference in hemoglobin (*p* = 0.007) between mild and severe groups. HCT was higher in moderate group (*p* = 0.003) compared to severe group. MCV was significantly increased (*p* = 0.012) in severe (108.59 ± 2.93) than the moderate group (93.63 ± 2.89). Neutrophil percentage was found to be significantly elevated (p = 0.002) in severe group (69.6 ± 2.16) compared to mild (54.7 ± 1.77). Similarly, lymphocyte percentage was significantly decreased (*p* = 0.001) in severe group (29.2 ± 1.94) with respect to mild (40.7 ± 1.68) and moderate (41.3 ± 1.42) groups. No significant predictive values of IQ scores (with age correction) were found on these hematological parameters after linear regression analysis. Significant correlations among the different hematological parameters are represented in Supplementary Table S9.

**Table 2:** The comparative assessment of different cellular parameters measured in three classified groups of ID children (mild, moderate and severe).

Parameters		Results
Oxidative stress related biomarkers	LPO of PBMC	Moderate > Mild = Severe
	GSH of PBMC	Mild = Moderate; Severe > Moderate
	SOD activity of PBMC	Mild > Moderate = Severe
	LPO of RBC membrane	Severe > Moderate > Mild
	GSH of RBC	Mild > Moderate > Severe
	SOD activity of RBC	Mild > Moderate > Severe
	Serum ROS level	Severe > Moderate > Mild
	Serum homocysteine level	Severe > Moderate > Mild
Neurotransmitters	Serum glutamate level	Severe > Moderate > Mild
	Serum GABA level	Mild > Moderate = Severe
	Serum serotonin level	Mild = Severe > Moderate
	Serum dopamine level	Mild > Moderate > Severe
Metabolic molecules	Serum lactate level	Severe > Moderate > Mild
	Serum LDH-A expression	Severe > Moderate > Mild
RBC structural protein	β-actin expression	Mild = Moderate = Severe
Neurotrophic factor	Serum BDNF expression	Mild = Moderate = Severe
Inflammatory molecules	TNF-α	Mild = Moderate = Severe
	IL-6	Mild = Moderate = Severe
Haematological parameters	Haemoglobin	Mild > Severe; Mild = Moderate
	HCT	Mild = Moderate > Severe
	MCV	Severe > Moderate = Mild
	Neutrophil	Mild < Moderate > Severe
	Lymphocyte	Mild > Moderate < Severe
Lipid profile	Triglyceride	Severe > Moderate > Mild
	Total cholesterol	Mild < Moderate > Severe
	LDL-C	Severe > Moderate > Mild
	HDL-C	Mild > Moderate > Severe
	VLDL-C	Severe > Moderate > Mild
Clinical and liver function related parameters	Urea	Mild = Moderate < Severe
	Globulin	Mild < Moderate = Severe
	A/G ratio	Mild > Moderate = Severe

Parameters are analyzed by ANCOVA (using age as the covariate) followed by correction for multiple testing (Bonferroni correction). The symbol “>” represents the level of one particular parameter in one group is greater compared to others. Conversely, the symbol “<” represents the level of one particular parameter in one group is lower compared to other two groups. Symbol “=” represents no statistical differences are found in between groups.

### Changes in clinical parameters

Fasting glucose, urea, creatinine, calcium, phosphorus, sodium, potassium, iron and CRP levels were analyzed in intellectually disabled children (see Supplementary Table S2). It was shown that serum urea level (mg/dl) exhibited significant increase (*p* = 0.003) in severe group compared to moderate which was influenced by age. However, no such correlation was found among these clinical parameters (see Supplementary Table S10).

### Changes in lipid profile

Serum lipid profile (Triglyceride, Total cholesterol, LDL cholesterol, HDL cholesterol and VLDL cholesterol) of disabled children was analyzed and changes in parameters among the ID categories were represented in Table 2. On performing appropriate statistical analyses followed by correction for multiple testing, we found that triglyceride (mg/dL) level was significantly elevated in severe group (161.8 ± 11.74) compared to mild (58 ± 3.35) and moderate (86.1 ± 3.18) individuals. Total cholesterol was increased in moderate group (151.8 ± 4.15) with respect to mild (114.2 ± 5.44) and severe (110.4 ± 3.18). LDL cholesterol level was found to be significantly increased in severe group (86 ± 3.37) compared to mild (60.7 ± 1.29) and moderate (76.7 ± 1.8) intellectually disabled subjects. HDL cholesterol was lower in severe (27.4 ± 2.09) and moderate (40.8 ± 1.83) groups than in mild (46.8 ± 2.78). VLDL cholesterol (mg/dL) was higher in severe group (38.4 ± 1.79) compared to mild (11.9 ± 0.79) and moderate (19.4 ± 1.52) ID groups. Significant predictive values of IQ scores (age adjusted) were found on triglyceride, LDL cholesterol, HDL cholesterol, VLDL cholesterol. Details of the correlation values among these parameters are provided in Supplementary Table S11.

### Differences in parameters associated with liver function in between three groups

Serum SGPT, SGOT, alkaline phosphatase, bilirubin, total protein, albumin (A), globulin (G), A/G were measured in ID individuals (Table 2). No significant differences were found among the parameters of intellectually disabled categories except serum globulin and A/G ratio (Supplementary Table S4). Globulin content was high [*p* value (ID groups) = 0.004; (age) = 0.001] in moderate group compared to the mild counterpart. Whereas, A/G ratio was higher [*p* value (ID groups) = 0.001; (age) = 0.001] in mild group compared to moderate. Strong correlations were found among total protein, globulin and A/G ratio (Supplementary Table S12).

### ID showed higher oxidative stress in RBC rather than PBMC

**Figure 2:**
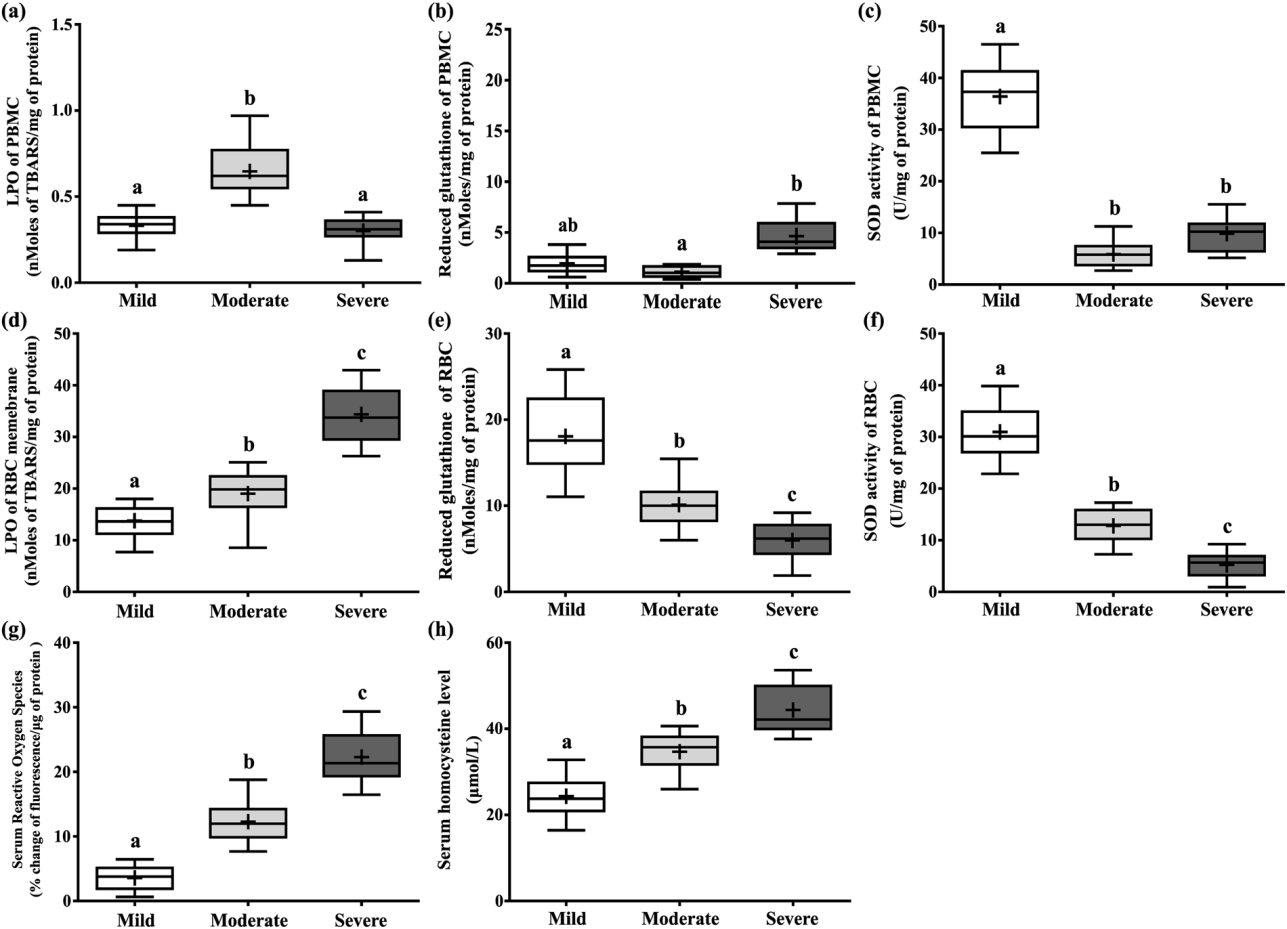
The status of oxidative stress related parameters were evaluated in the three ID groups. (**a**) TBARS formation of PBMC (nMoles of TBARS/mg of protein), (**b**) Levels of reduced glutathione of PBMC (µMoles/mg protein), (**c**) SOD activity of PBMC (Unit activity/mg protein), (**d**) TBARS formation of RBC membrane (nMoles of TBARS/mg of protein), (**e**) Levels of reduced glutathione of RBC (µMoles/mg protein), (**f**) SOD activity of RBC (Unit activity/mg protein), (**g**) Serum ROS level using H_2_DCFDA. In the diagram, the bottom line, top line and line in the middle of each box represents 25th percentile, 75th percentile and 50th percentile (median) distribution of scores respectively. The lower and upper whiskers represent 5% and 95% CI values of each group respectively. Plus sign (+) in each box represents the mean value of each group. Parametric values are represented as Mean ± SEM and significance between the groups were evaluated by performing ANCOVA (using age as the covariate) followed by correction for multiple testing (Bonferroni correction). The letters (a, b, c) stand for significant differences in parametric values of mild, moderate and severe Intellectual disabled groups. The similar letters designate without any significant differences between groups. The letters ‘ab’ indicate that the value is not significantly different compared to either ‘a’, or ‘b’.

The purpose of determination of oxidative stress markers as well as antioxidant status was to evaluate whether it was associated with progression of disease severity or not (Fig. 2). ROS plays an important role in mediating OS. Oxidative stress signature molecules were determined from isolated PBMC and RBC of different ID groups. Extent of lipid peroxidation in terms of TBARS formation, glutathione level and SOD activity were analyzed both from PBMC and RBC. Lipid peroxidation (nMoles of TBARS/mg of protein) was found to be increased significantly in PBMC of moderate group (0.55 ± 0.04) of ID compared to mild (0.38 ± 0.03) and severe (0.31 ± 0.03) (Fig. 2a). Glutathione level in PBMC (nMoles/mg of protein) found significantly reduced in moderate group (1.6 ± 0.09) with respect to severe (2.98 ± 0.22) (Fig. 2b). SOD activity of PBMC (U/mg of protein) was reduced in moderate (5.38 ± 0.51) and severe groups (8.43 ± 0.7) compared to mild (36.73 ± 2.06) (Fig. 2c). Significant influence of IQ scores (age adjusted) on SOD activity of PBMC (*p* = 0.001) was found after linear regression analysis (see Supplementary Table S6).

LPO (nMoles of TBARS/mg of protein) of RBC membrane has increased with the severity of ID. TBARS formation of RBC membrane has increased in moderate (20.35 ± 0.63) and severe (29.21 ± 1.44) with respect to mild group (15.68 ± 0.54) (Fig. 2d). Glutathione level (nMoles/mg of protein) of RBC was found to be lower in moderate (9.84 ± 0.28) and severe group (8.14 ± 0.27) compared to mild (14.91 ± 0.94) (Fig. 2e). SOD activity (U/mg of protein) of RBC has reduced in moderate (14.43 ± 0.82) and severe (6.79 ± 0.5) groups with respect to mild one (29.08 ± 1.89) (Fig. 2f). Significant association of IQ values (age adjusted) was found on TBARS formation, GSH and SOD activity among the respective ID categories (see Supplementary Table S6).

Serum ROS level (% change in fluorescence/µg of protein) was significantly higher in moderate (10.41 ± 0.57) and severe (19.99 ± 0.66) compared to the mild group (4.51 ± 0.3) of ID children (Fig. 2g). From pair-wise comparison it was shown that Hcy level (µmol/L) was significantly increased in severe group (42.09 ± 1.67) with respect to mild (26.06 ± 1.42) and moderate (36.51 ± 0.87) (Fig. 2f) ID groups. Significant association of IQ values (age adjusted) on ROS and Hcy was found after performing linear regression (see Supplementary Table S6).

The detailed results of the correlation study among these oxidative stress biomarkers were provided in Supplementary Table S13.

### Changes in serum neurotransmitter levels

**Figure 3:**
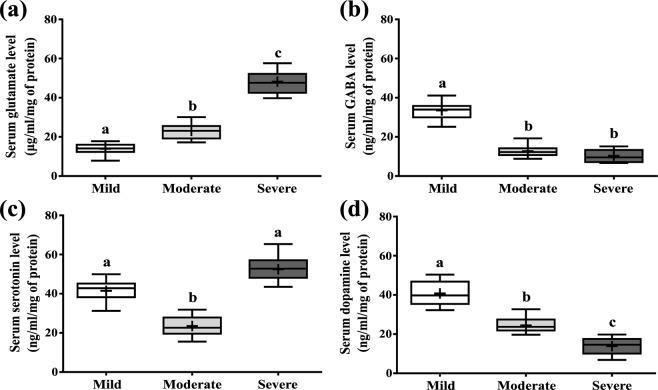
Changes in serum neurotransmitters in the three groups of ID using ELISA. (**a**) Serum glutamate (μg/ml/mg of protein), (**b**) serum GABA level (ng/ml/mg of protein), (**c**) Serotonin level (ng/ml/mg of protein), (**d**) Serum dopamine (ng/ml/mg of protein). In the diagram, the bottom line, top line and line in the middle of each box represents 25th percentile, 75th percentile and 50th percentile (median) distribution of scores respectively. The lower and upper whiskers represent 5% and 95% CI values of each group respectively. Plus sign (+) in each box represents the mean value of each group. Parametric values are represented as Mean ± SEM and significance between the groups were evaluated by performing ANCOVA (using age as the covariate) followed by correction for multiple testing (Bonferroni correction). The letters (a, b, c) stand for significant differences in parametric values of mild, moderate and severe Intellectual disabled groups.

Serum glutamate, GABA, serotonin and dopamine were estimated from serum of intellectually disabled children (Fig. 3). Serum glutamate, GABA, serotonin and dopamine levels differ remarkably in three ID groups (Table 2). After pair-wise comparison between the groups, we found that glutamate level (µg/ml/mg of protein) was significantly higher in severe group (47.82 ± 1.76) compared to mild (14.54 ± 0.84) and moderate group (22.92 ± 1.06) (Fig. 3a). Alternatively, serum GABA (ng/ml/mg of protein) and dopamine (ng/ml/mg of protein) were found to be lower with increasing severity of disability. GABA has reduced significantly in severe (10.03 ± 0.85) and moderate group (12.51 ± 0.79) with respect to mild one (31.79 ± 0.95) (Fig. 3b). In a similar manner, serum dopamine level has reduced significantly in moderate (23.83 ± 0.45) and severe group (16.12 ± 0.67) than the mild (33.43 ± 1.22) group (Fig. 3d). Serotonin (ng/ml/mg of protein) was reduced significantly in moderate group (28.85 ± 1.31) compared to mild (40.28 ± 0.51) and severe groups (49.27 ± 1.31) (Fig. 3c). Significant influence of IQ values (age adjusted) was found on glutamate level after regression analysis (see Supplementary Table S7). The values of correlation tests among these neurotransmitters are represented in Supplementary Table S14.

### Increase in serum lactate and LDH-A expression with the severity of ID

**Figure 4:**
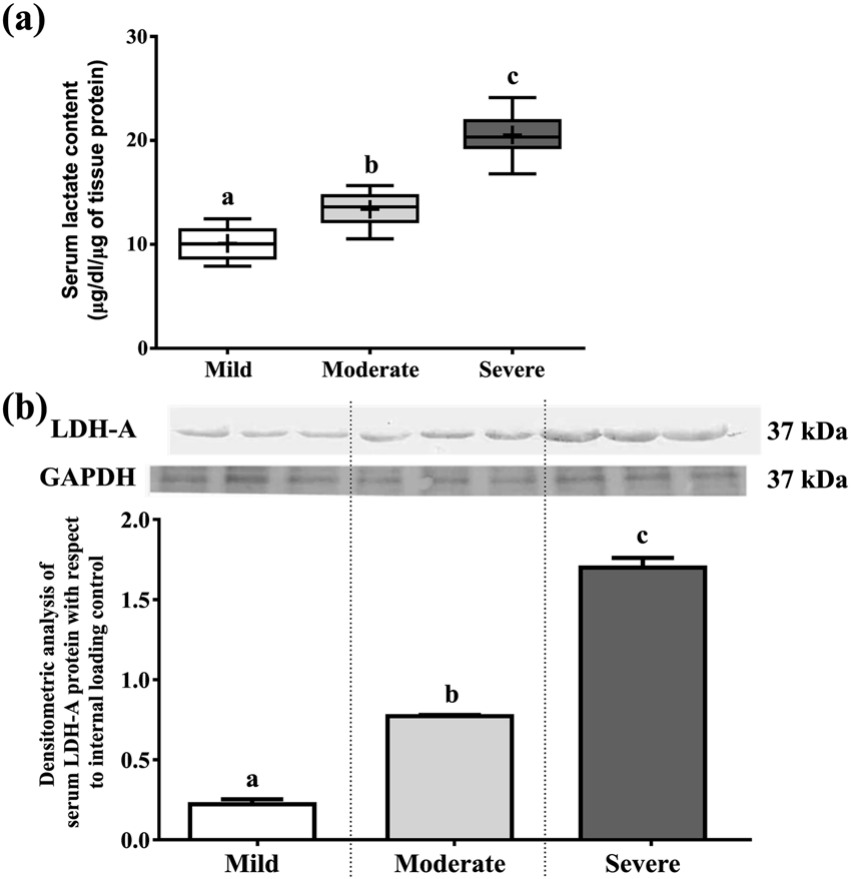
The status of serum lactate content and LDH-A expression of three ID groups. (**a**) Serum lactate content is measured using spectrophotometer, (**b**) Immunoblot analysis is performed using antibody specific for LDH-A from serum using GAPDH as internal loading control. Relative intensity is obtained from densitometric analysis of the respective immunoblot. Plus sign (+) in each box represents the mean value of each group. Values were represented as Mean ± SEM and significance between the groups were evaluated by performing ANCOVA (using age as the covariate) followed by correction for multiple testing (Bonferroni correction). The letters (a, b, c) stand for significant differences in parametric values of mild, moderate and severe Intellectual disabled groups. The similar letters designate without any significant differences between groups. The letters ‘ab’ indicate that the value is not significantly different compared to either ‘a’, or ‘b’.

Lactate dehydrogenase (LDH) and lactate are some of the hypoxy biochemical parameters. Extracellular activity of LDH elevates under certain conditions like oxidative stress, since the cell integrity can be disrupted during the lipid peroxidation process. This leads to increased lactic acid and lactic acid salts. Lactate level (µg/dl/µg of protein) was significantly increased in severe (20.54 ± 0.67) with respect to mild (10.09 ± 0.47) and moderate group (13.38 ± 0.5) (Fig. 4a). LDH-A expression was up regulated in severe group (0.24 ± 0.02) than mild (0.11 ± 0.02) and moderate (0.15 ± 0.01) groups of individuals (Fig. 4b). Strong association of IQ scores on lactate and LDH-A were found after linear regression analysis. Strong positive correlation was found (*p* < 0.001) between lactate and LDH-A (see Supplementary Table S15).

### RBC morphology was altered in intellectually disabled children

**Figure 5:**
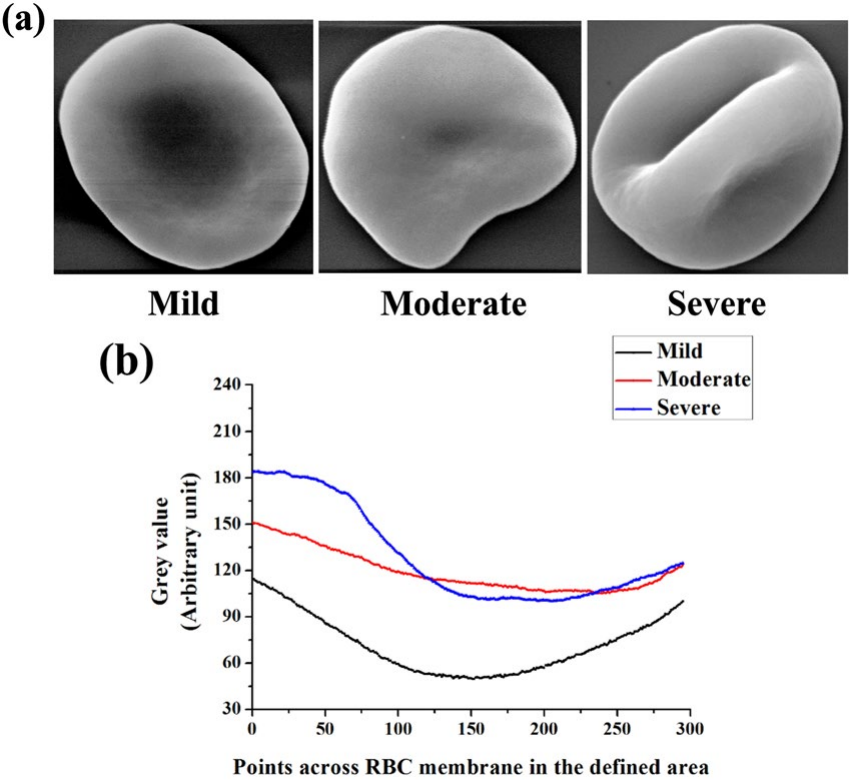
Scanning electron microscopy images of RBC from (**a**) Mild, moderate and severe disabled subjects. Scale- 1 μm, magnification: 25,000 × machine magnification. (**b**) Quantitative representation of RBC surface topology showing different alterations in mild (black line), moderate (red line) and severe (blue line) ID groups.

Figure 5a shows altered image of RBCs of the three groups (25000 × machine magnification). There was change in RBC structure between the groups of intellectually disabled children and the deviation from its normal morphology was found to be more with increasing severity of disability. General shape alterations were found in erythrocytes of ID from its typical discoid appearance. The surface topologies were analyzed and represented in Fig. 5b.

### Representation of β-actin of RBC across severity of disability

**Figure 6:**
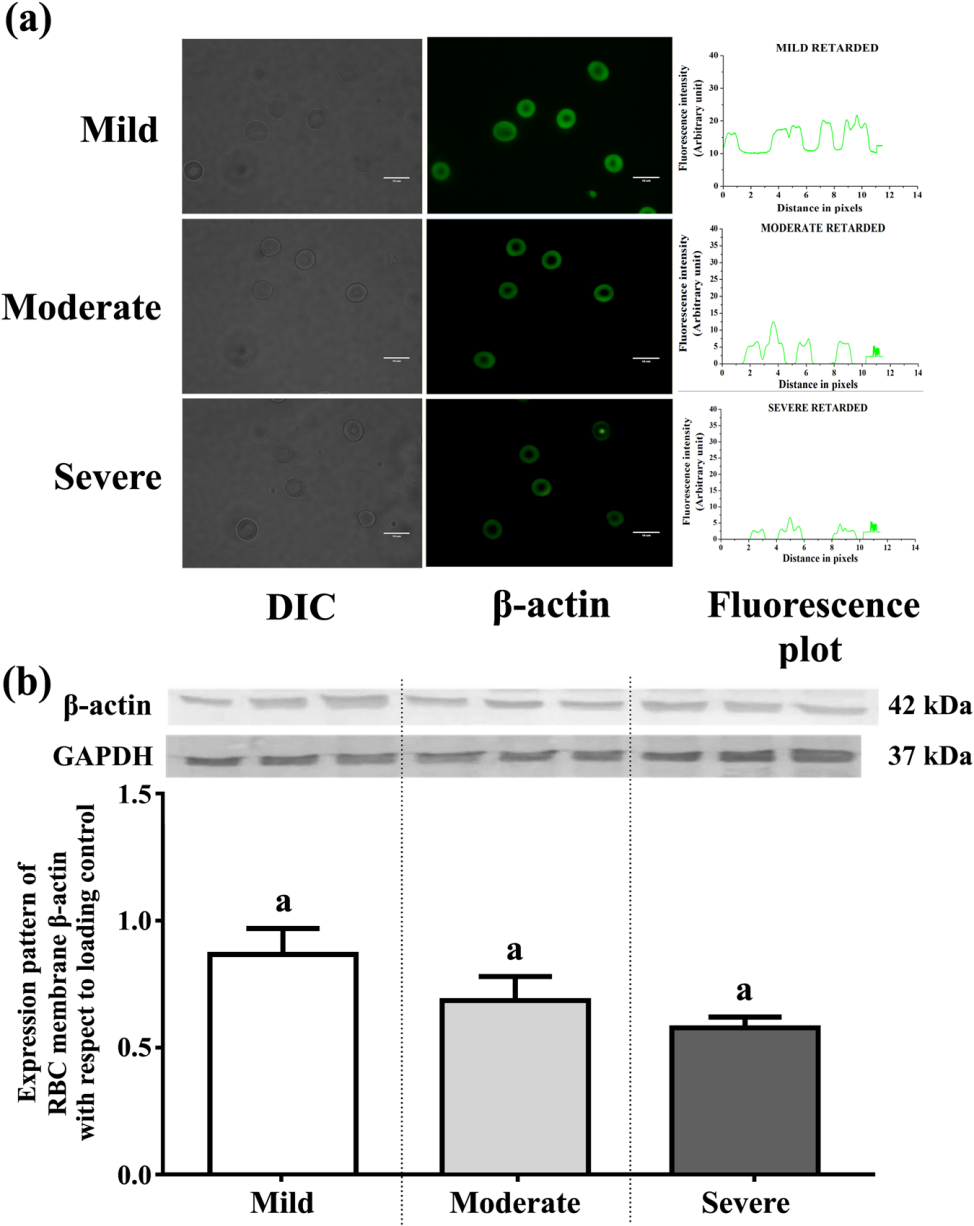
(**a**) Cellular localization and expression levels of β-actin of RBC of three ID groups (mild, moderate and severe). RBCs are stained for β-actin expression using FITC. Scale bars: 50 mm. Images are acquired by FITC fluorescence channel, using suitable filters with 60X objective. Each graphical representation is analyzed from the respective micrographs using ImageJ software. (**b**) Expression pattern of RBC membrane β- actin of ID groups. Immunoblot analysis was performed using antibody specific for β-actin and GAPDH is used as internal loading control. Relative intensity is obtained from densitometric analysis of the respective immunoblot. Values were represented as Mean ± SEM and significance between the groups were evaluated by performing ANCOVA (using age as the covariate) followed by correction for multiple testing (Bonferroni correction). The letters (a, b, c) stand for significant differences in parametric values of mild, moderate and severe Intellectual disabled groups. The similar letters designate without any significant differences between groups.

Erythrocyte membrane integrity is critical for maintaining the characteristic shape of erythrocyte and is based on both vertical and horizontal interactions among the cytoskeletal proteins, the integral membrane proteins, and the phospholipid bilayers. To determine the changes in RBC cytoskeletal protein expression with the severity of ID, we examined the expression level of β-actin from RBC by Immunofluorescence study. Results showed that β-actin expression apparently decreased with increasing degree of ID (Fig. 6a). Expression was further validated by determining expression of β-actin from RBC membrane fractions of the three groups using Immunoblot technique (Fig. 6b). However, we did not find any significant change in between ID groups after appropriate statistical analyses with age correction (Supplementary Table S8).

### Representation of pro-inflammatory proteins and a neuro-modulator with severity of ID

**Figure 7:**
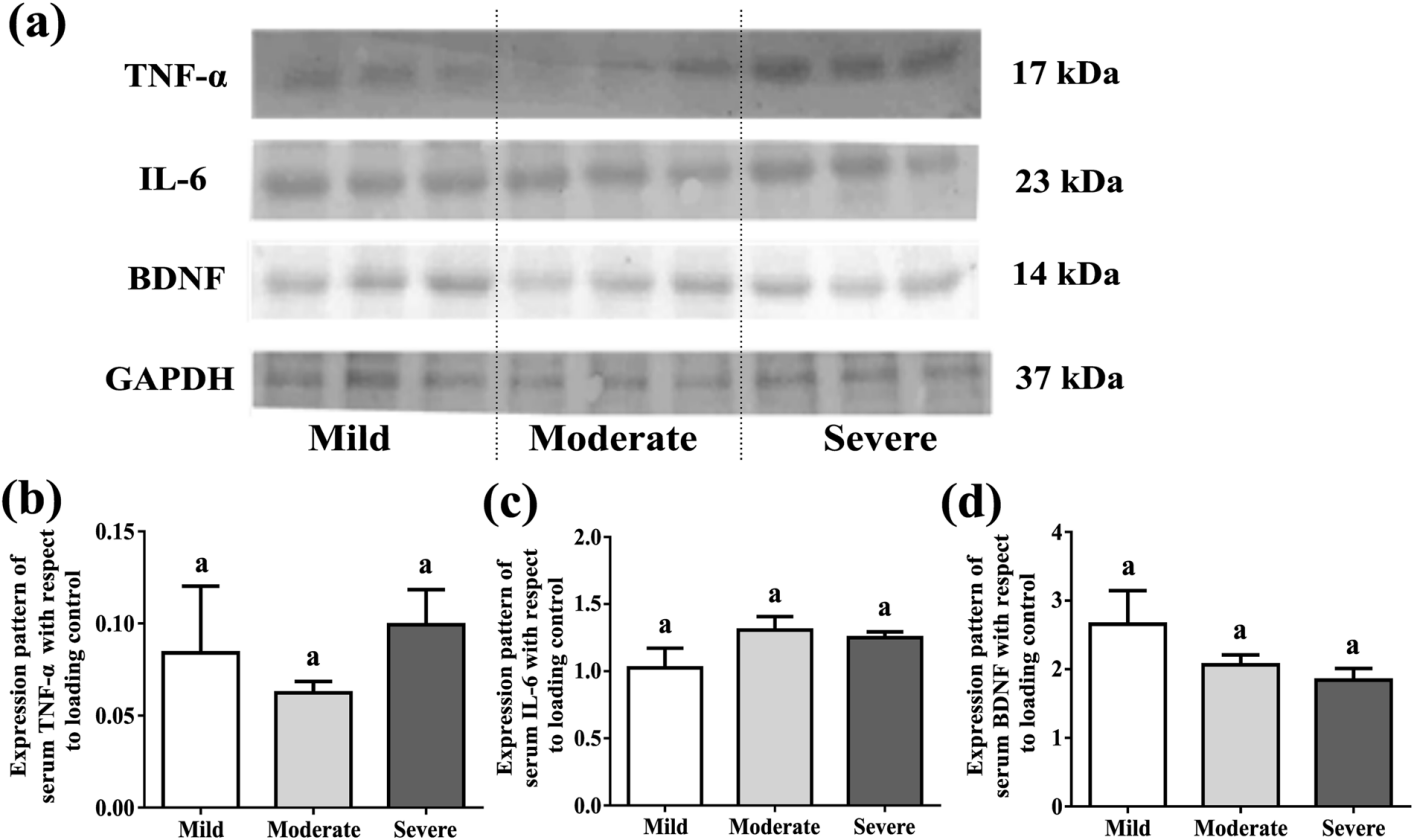
Expressions of serum pro-inflammatory markers and neurotrophic molecule in ID groups. (**a**) Cropped immunoblot images of TNF-α, IL-6 and BDNF. GAPDH was used as loading control. (**b**) Relative intensity is obtained from densitometric analysis of the respective immunoblot serum TNF-α, IL-6 and BDNF. Values were represented as Mean ± SEM and significance between the groups were evaluated by performing ANCOVA (using age as the covariate) followed by correction for multiple testing (Bonferroni correction). The letters (a, b, c) stand for significant differences in parametric values of mild, moderate and severe Intellectual disabled groups. The similar letters designate without any significant differences between groups.

There are reports showing that serum pro-inflammatory cytokines are dysregulated in patients with intellectual disability. Expressions of TNF-α, IL-6 and BDNF were unaltered with increasing severity of disability after age adjustment (Fig. 7a–d). The details of the statistical analysis are provided in Supplementary Table S8.

## Discussion and Conclusion

The onset of development of intellectual disability (ID) cannot be prevented. At this stage, the causative factors for disease progression also remain unalterable in a significant way. However, with the advanced knowledge and analysis, we can offer early diagnosis and hence a better management to limit the progression in those individuals who cannot manifest independent participation and social adaptations. Thus, determining the true modifiable indicators of pathogenesis and association between them were the chief objectives of this current study. Therefore, the present article for the first time has extended a wide range of unique insights on how severity of disability is correlated with psycho-physiological, cellular and molecular biomarkers.

To our knowledge, the physiological and cellular changes with the degree of severity of intellectual disability have not been reported previously. For many persons with ID, it is unclear whether a genetic cause or an unknown exogenous cause is responsible for the pathophysiology. In our current piece of work, we identified the gross deficit of psycho-social constituents (MA and SA) of the ID individuals with the severity of the disease (Fig. 1). Thus, intellectual growth and social functioning is found to be affected in mentally challenged individuals, which is primarily associated with degree of impairment^2^.

Low hemoglobin is often associated with cognitive deficits, decline in semantic memory and perceptual speed. In this study, higher MCV values might have influenced the RBCs to become macrocytic in nature. It is likely that vitamin B_12_, folate deficiency, myeloplastic syndromes, reticulocytosis, inflammation, or excessive oxidative stress also can be the reasons behind such clinical manifestation^22^. Moreover, increased HCT value in severe group may implicate insufficiency in oxygen concentration or impaired oxygen saturation in blood. Such systemic dysfunction can results in impaired neuronal energetic metabolism and ultimately cognitive dysfunctioning.

Several studies have demonstrated the linkage between disease severity and defects in antioxidant defense system. In our case both enzymatic and non-enzymatic antioxidant defense of RBC gradually declined with increasing severity of ID (Fig. 2d–f). The altered activities of RBC antioxidant enzymes in disabled children might serve as a peripheral response of the entire system for increased free oxygen radical production in the CNS. We preferred the findings from erythrocytes to estimate the status of systemic OS, because these antioxidant enzymes are constitutively expressed in the first stages of its maturation process.

The maintenance of the discoid shape of red cells is of paramount importance for maintaining the main physiological role. The deformed circulating RBCs lead to difficulty in passing through capillaries and transporting adequate quantities of oxygen and nutrients to tissues. Our observations indicated that abnormal erythrocyte shapes were predominant in the peripheral blood of individuals with intellectual disability (Fig. 5). In the brain, such systemic dysfunction can impair neuronal energy metabolism and, in turn; negatively affect cognitive functioning^23^. The presence of abnormal erythrocyte shapes appeared to be associated with an altered redox status and oxidization of the important cytoskeletal proteins such as β-actin known to be critical for the maintenance of the horizontal interactions in the erythrocyte membrane architecture. The detection of reduced fluorescence intensity of RBC membrane β-actin is in agreement with the other studies documenting cytoskeletal defects in neuro-developmental diseases (Fig. 6a).

The OS markers in PBMC did not follow the similar trend like in RBCs. We found differential patterns of OS related parameters in case of PBMC (Fig. 2a–c). Serum total ROS level also elevated with increasing severity of ID (Fig. 2g). H_2_O_2_ is a neutral and highly liposoluble substance and it can easily pass through the cytoplasmic membranes. Therefore, an excess amount of H_2_O_2_ produced in or outside the blood cell membrane (RBC, PBMC and platelet) may pass through the cell membrane into the serum^24^. Our findings denoted that elevated serum ROS was positively correlated with increased lipid peroxidation of RBC and negatively correlated with RBC antioxidants and SOD activity PBMC (see Supplementary Table S13). All these findings together lend further support to the concept that an increased vulnerability to oxidative stress may contribute to the development of manifestation of ID.

Down the line, Homocysteine (Hcy) possibly induces oxidative stress (generation of H_2_O_2_) by auto oxidation of sulfhydryl group (-SH) of homocysteine and decreases intracellular antioxidant defense (glutathione and glutathione peroxidase) mechanisms, induces apoptosis, trigger mitochondrial dysfunction and ultimately leads to cellular damage in various organisms^25^. Present article reports that the serum Hcy level was significantly associated with the severity of intellectual disability (see Supplementary Table S6). Elevated level of plasma Hcy is associated with cognitive impairment and atrophic changes in the brain which is widely observed among neuro-psychiatric patients including affective disorders and schizophrenia^26^. Thus, elevated serum Hcy might be a consequence, rather than a cause of oxidative stress and a reflection of low vitamin B_12_ and folate in the system.

The metabolic stress was further confirmed by increased serum lactate level and LDH-A protein with increasing severity of disability (Fig. 4). Some findings suggested that autistic patients often suffer from lactic acidosis and hyperpyruvatemia that may be due to defects in carbohydrate metabolism and/or mitochondrial disturbances^27^. High serum LDH were found in patients with mania, depression, schizo-affective or atypical psychosis compared to non-psychiatric control subjects^28^. In the present study, the rise of serum lactate in severe group was positively correlated with higher LDH-A expression (see Supplementary Table S15).

Elevated Hcy level can augment the cellular stress, which in turn activate many metabolic perturbations including the lipid biosynthesis pathway. This metabolic impairment was one of the predictors of disabled population having increased risk of obesity, diabetes and cardiovascular disease^29^. In this study, we found strong association among dysregulation of the serum lipid profile (except total cholesterol) and progression of disability (see Supplementary Table S3). The dyslipidemic conditions were developed more in severely affected individuals may be due to impairment in lipid metabolism or physical inactivity of the children.

The neurotransmitters manifested crucial picture of the disability. The present study truly indicated the reciprocal relationship of higher serum level of glutamate and lower levels of GABA and dopamine across the severity groups (Fig. 3a,b,d). On the contrary, differential pattern of serotonin was found with increasing degree of severity (Fig. 3c). Glutamate is involved in neuro-inflammation in patients with neurodevelopmental diseases^30^. Glutamate, essentially being an excitatory NT, its higher level in severe groups explains aggression of the ID subjects. Moreover, the higher levels of Hcy justified this finding as it interacts with NMDA receptors and promulgate the neuro-toxic effect of excess glutamate. Other experiments on different neuro-psychiatric models substantiate our current findings, however, the exact molecular regulation of neurotransmitters remain as an enigma.

Neutrophil-lymphocyte ratio (NLR) is reported in recent studies as a peripheral inflammation marker in several neuropsychiatric disorders^21^. Thus we thought that it could also represent the systemic inflammatory status in part. We observed that percentage of neutrophil increased in severe group of ID subjects whereas percentage of lymphocyte decreased with severity. To confirm our findings further, the pro-inflammatory cytokine expressions were determined. However, after age adjustment, their expressions remain non-significant across severity groups. Moreover the BDNF expression remains unaltered after age adjustment though there were many clinical evidences present suggesting its reduction in many psychiatric and neuro-developmental diseases^31^.

In recent past, there were few impactful studies which focused on independent predictors of disability. However, the present study with unique cellular and inflammatory biomarkers, neurotransmitters and other signature molecules is a comprehensive one (see Supplementary Fig. S1). These related biomarkers might be advocated as strong predictors of severity of disability.

The major goal of this study was to establish association between the parameters with the three classified groups of the ID subjects. We found strong association of raw IQ scores with hematological parameters, lipid profile, oxidative stress parameters and neurotransmitters. However, we would like to point out certain caveats in our findings. A larger sample size would of course have resulted in higher statistical power and interpretation of the findings. Therefore, due to small sample size, there is an ample chance for reflection of differences between the respective parameters other than the IQ score. The study is cross-sectional in nature. The cross-sectional nature of the study also prevents us from investigating the change in blood parameters with time as we collected the blood from the same subjects for number of occasions. We did not perform any genetic and epigenetic analyses of the samples and as such could not determine the onset of pathophysiology. Nonetheless, this was intended to be a baseline study for early assessment of probable blood indicators associated with severity of disease. More rigorous and intensive studies are required in the future to establish the facts more stringently. The current piece of work might be highly beneficial for effective clinical outcome in favor of ID children. This study is ideal as a model for identification of predictors and establishment of treatment paradigm for this neglected part of the society. Therefore, some of the lifestyle alterations like regular yogic and physical exercises may limit the aggravation of the disability.

## Materials and Methods

Histopaque-1077, Trichloroacetic acid (TCA), Thiobarbituric acid (TBA), 5,5′-dithio-bis (2-nitro benzoic acid) (DTNB), acrylamide-bis acrylamide, Tween 20 were purchased from Sigma Aldrich (St. Louis, MO, USA). 2′, 7′-dichlorodihydrofluorescein diacetate (H_2_DCFDA), pyrogallol, H_2_O_2_, ethanol and all other fine chemicals were procured from Merck (Germany). Glutamate ELISA kit was purchased from Abnova (Taiwan). GABA, serotonin and dopamine kits were purchased from QAYEE-BIO (Shanghai, China). Homocysteine ELISA kit was procured from Randox (Antrim, United Kingdom). All antibodies were purchased from Cell Signaling Technology (Danvers, MA, USA) and other chemicals used were of highest purity grade available.

### Ethical approval

The approval from the Institutional Human Ethics Committee, Department of Physiology of University of Calcutta (Ref no. IHEC/SD/P29/13, dated 22.03.2013) was obtained for this work and appropriate measures were taken to follow all ethical norms throughout the study. An information sheet describing the rationale of the study and individual rights was handed to the care givers of the participants. Written informed consents were then obtained from the legally authorized representatives. All experiments were performed in accordance with the approved guidelines and regulations of Human Ethics committee and the Institutional Bio-Safety Committee of University of Calcutta. All the procedures involving the subject handling was done under the supervision of an experienced neuro-physician.

### Human Subject Selection

All studies were performed in randomly selected intellectually disabled children [Male, Total number of subjects (N) = 45, Number of subjects in individual groups (n) = 15, age 10 ± 5 years]. The pathophysiology of the disease was diagnosed following the guidelines of Diagnostic and Statistical Manual of Mental Disorders: Fourth Edition-V (DSM-V) (2013), Washington, DC: American Psychiatric Association. Purposive sampling procedure was followed from Government aided NGO-run rehabilitation homes with ID inhabitants. The subjects were then categorized based on their IQ scores using standard psychometric test.

### Inclusion criteria

The age ranges of the subjects were selected from 10 ± 5 years. Male subjects were chosen for these experiments.

### Exclusion criteria

Individuals with anemia, other blood related diseases, HIV, Hepatitis B and C or already involved in any other clinical trials were not considered for this study.

### Psychological Tests

The psychological tests were performed in presence of registered Psychologist and Experienced Practitioner.

### Measurement of intelligence quotient (IQ) and mental age by Stanford Binet Intelligence Test

It is a cognitive ability assessment used to measure intelligence (IQ). The Stanford-Binet measures five factors of cognitive ability: Fluid Reasoning, Knowledge, Quantitative Reasoning, Visual-Spatial Processing, and Working Memory. Every factor was tested in two separate domains, verbal and nonverbal^32^. For this test, IQ levels for mild were considered as 50–70, moderate 35–49 and severe 20–34.

### Determination of social age using Vineland Social Maturity Scale (VSMS)

The Vineland Social Maturity Scale is a psychometric assessment instrument that is useful for the assessment of social competence. It is a psychometric questionnaire and measures social maturity or social competence in people from birth to adulthood. It is classified into eight categories of items on the VSMS: self-help general, self-help dressing, self-help eating, communication, self-direction, socialization, locomotion, and occupation^33^.

### Blood collection

Whole blood (10 ml) was collected from the individuals. Serum, peripheral blood mononuclear cells (PBMC) and RBC were isolated from the whole blood by a density gradient technique using Histopaque-1077^34^.

### Isolation of PBMC

PBMC were immediately isolated from fresh blood by density gradient centrifugation according to the standard protocol. Briefly, 5 ml blood was layered carefully over an equal volume of Histopaque-1077 and then centrifuged for 30 min at 400 × g. PBMC were collected from the buffy coat formed at the plasma–Histopaque-1077 interface and the pellet was re-suspended in PBS (50 mM, pH 7.4). This process was repeated twice or thrice to remove the platelets^35^.

### Preparation of cell lysate of PBMC

Isolated PBMC (8 × 10^6^) was suspended in 100 μl hypotonic buffer (1.5 mM MgCl_2_, 10 mM KCl, 1 mM dithiothreitol, 10 mM HEPES, pH = 7.9) containing protease inhibitor cocktail and sonicated. The suspension containing the lysed cells was centrifuged at 13,000 × g for 10 min at 4 °C. The supernatant (cell homogenate) was then used for antioxidant enzyme assays and western blot analysis^35^.

### Preparation of RBC for antioxidant assay

Blood samples were drawn into glass tubes containing Na-ethylenediaminetetraacetic acid (EDTA). RBCs were separated from plasma by centrifugation at 700 × g at 4 °C for 15 minutes and washed 3 times with 0.9% saline solution with removal of the buffy coat^36^. Aliquots of the RBCs were taken for determination of GSH and SOD.

### Isolation of RBC membrane

Freshly drawn blood was used for membrane preparation. The RBC membrane was prepared using a standard protocol with minor modification^37^. Briefly, hypotonic phosphate buffer was added to the suspension of erythrocyte and centrifuged at 25,000 × g for 40 min. Red loosely packed membrane pellet was resuspended in hypotonic phosphate buffer and centrifuged at 20,000 × g for 20 min. This step was repeated five to six times more till a milky-looking membrane pellet was formed and it was re-suspended in PBS (pH 7.4). This was used for determination of LPO and Western blot analysis.

### Analysis of clinical parameters

Parameters such as percentage of hemoglobin (Hb), CRP, ESR, glucose (random), urea, creatinine, total lipid profile, liver function tests, mineral content in blood were determined using auto-analyzer in all three ID groups (N = 45) (Purechem Ltd, Ireland).

### Protein estimation

Protein content of serum, PBMC and RBC were determined by Bradford method using BSA as a standard^38^.

### Determination of LPO

The lipid peroxidation of RBC and PMBC membrane was estimated using standard protocol of formation of TBARS in the sample. In brief, the cell fractions (PBMC lysate and RBC membrane) were mixed with 15% TCA, 0.375% TBA and 5(N) HCl and incubated for 15 min at 95 °C. After cooling, the mixture was centrifuged at 3000 × g for 10 min at room temperature and the absorbance of supernatant was measured spectrophometrically (Bio-RAD SmartSpec^TM^Plus Spectrophotometer) at 535 nm against appropriate blank^39^.

### Determination of GSH

Cell fractions (RBC and PBMC in respective cases) were mixed with 0.1 ml of 25% TCA and the mixture was centrifuged at 3,900 × g for 10 min in room temperature. The supernatant was collected in another tube and GSH level of the supernatant was assayed in a total reaction mixture of 3 ml [2 ml of 0.5 mM DTNB prepared in 0.2 M phosphate buffer (pH 8.0), with 1 ml of the supernatant]. The absorbance of resultant yellow complex was measured spectrophotometrically (Bio-RAD SmartSpec^TM^Plus Spectrophotometer) at 412 nm^36^.

### Determination of SOD activity

The activity of SOD in RBC and PBMC was determined using modified pyrogallol auto-oxidation method^40^. In brief, 62.5 mM tris-cacodylic acid buffer was mixed with cell fractions (RBC and PBMC in respective cases) followed by addition of 4 mM pyrogallol. The auto-oxidation of pyrogallol was monitored spectrophotometrically (Bio-RAD SmartSpec^TM^ Plus Spectrophotometer) at 420 nm, followed by the estimation of the absorbance of the test samples at specific time intervals.

### Determination of serum reactive oxygen species (ROS) level

The serum ROS level was determined by spectrofluorometric method using 2′-7′-dichlorofluorescein-diacetate (H_2_DCFDA) dye^41,42^. In brief, 15 µg of serum protein was mixed with 10 µl of 100 µM H_2_DCFDA, and PBS (pH 7.4) in a total reaction volume of 50 µl and the reaction mixture was incubated for 30 min at 37 °C in a water bath. For blank, 10 µl of 100 µM H_2_DCFDA was added to the PBS in a total reaction volume of 50 µl and DCF formation was monitored after 30 min incubation at 37 °C in order to subtract background auto-fluorescence values. The reaction was stopped by adding equal volume of ice-cold PBS in the reaction mixture and the fluorescent DCF (oxidized form) was measured in spectrofluorimeter (JASCO, India) using excitation and emission wavelengths at 495 nm and 525 nm, respectively. The entire experiment was performed in a dark room. Serum ROS level was expressed as percentage change of fluoroscence (DCF)/µg of protein.

### Determination of serum lactate

The serum lactate level was measured according to the manufacturer’s instructions (Bio-vision Inc., USA)^43^. Serum containing 50 µg of protein was pipetted into 1.5 ml micro centrifuge tube. The reaction volume of the micro centrifuge tube was adjusted to 256.25 µl (final reaction volume) with PBS (pH 7.4) and 100 µl of color reagent, then the entire solution was mixed well by vortexing. The reaction mixture was incubated for 10 minutes at 37 °C in water bath and the reaction was stopped by adding 244.75 µl of ice cold PBS (pH 7.4) in it. The absorbance was measured in spectrophotometer (UV-1800 UV-VIS, Shimadzu) at 570 nm against a reagent blank prepared from 400 µl of PBS (pH 7.4) and 100 µl of color reagent. The lactate content of the serum was expressed as µg/dl/µg of protein.

### Glutamate assay

The level of glutamate was measured according to the manufacturer’s instructions (Abnova, KA1670, Taipei, Taiwan) provided with the ELISA kit^12^.

### Determination serum GABA, serotonin and dopamine level

The level of GABA, serotonin and dopamine and were measured according to the manufacturer’s instructions (QAYEE-BIO, Shanghai, China) by ELISA kit method^44–46^.

### Detection of Homocysteine (Hcy) level

The tissue Hcy level in serum was measured according to the manufacturer’s instructions in auto-analyzer instrument setup (RX Daytona, Randox, Crumlin, Antrim, UK)^10^.

### Scanning Electron microscopy of RBC

RBC morphology was studied on whole blood smears using scanning electron microscope (SEM) following slightly modified the protocol^35^. Whole blood was diluted 1:1 with phosphate buffered saline (PBS, pH 7.4) and placed it at 37 °C for 10 min. Samples were centrifuged three times at 1,500 rpm for 5 min before they were fixed in 2.5% glutaraldehyde in Dulbecco’s phosphate buffered saline (DPBS) solution with pH of 7.4 for 60 min. The samples were rinsed and washed with phosphate buffer three times for 5 min before being fixed with 1% osmium tetra-oxide (OsO4). This was followed by another three times rinsing with PBS for 5 min each time, followed by a serial dehydration in 30%, 50%, 70%, 90% ethanol and three times with 100% ethanol. A smear of the preparation was prepared on a glass cover slip, dried, mounted and coated with platinum. An EVO-18 special edition SEM system (ZEISS, Germany) was used to study the morphology of erythrocytes.

### Immunofluorescence study of RBC

1 ml of each blood sample was centrifuged at 800 × g for 10 minutes at 4 °C and washed twice with PBS for Immunofluorescence study of RBC. The packed cells were smeared onto grease free glass slides and after drying were fixed with 4% paraformaldehyde dissolved in PBS at room temperature for 10 min. It was then permeabilized with 0.1% Triton X-100 in PBS for 5 minutes at 4 °C and washed three times with PBS. Cells were blocked with 1% BSA for 30 min at room temperature and incubated overnight with mouse monoclonal β-actin primary antibody (1:50 dilution in PBS). Following washes in TBS, cells were incubated with specific rabbit anti-mouse FITC tagged secondary antibody (1:100 dilution in PBS) for 1 hr at room temperature. Finally, the fluorescent RBCs were washed followed by covering with mounting solution and visualized using the Olympus confocal microscope (Shinjuku, Tokyo, Japan). The relative fluorescence intensities of β-actin of RBC from three ID groups were estimated from the intensity curves, and each immunofluorescence image was analyzed by ImageJ software^9^.

### Western blot analysis

40 µg of protein (for serum cytokines) and 50 µg of protein for RBC membrane were loaded in each lane on 12.5% SDS-PAGE and transferred it onto a PVDF membrane. After blocking with 5% bovine serum albumin (BSA) in TBST for 1 hr, the membranes were incubated with primary antibody (1:1500 dilution with TBST) for over-night, followed by incubation with secondary antibody (1:2000 dilution with TBST) for 2 hrs at room temperature. Protein bands were visualized using NBT-BCIP solution mixture. The relative protein levels were calculated by normalization to the amount of internal control protein. Polyclonal antibodies were used for TNF-α and IL-6 (Imegenex, San Diego, CA, USA) and monoclonal for β-actin, GAPDH, LDH-A (Cell Signalling Technology, Danvers, MA, USA), BDNF (Abcam, Cambridge, United Kingdom). GAPDH was used as loading control^47,48^.

### Statistical analysis

Statistical analysis of the data was performed using SPSS Statistics, version 23 (IBM Corporation, Armonk, NY). As we found variations in the age range between the three ID groups, we carried out a one-way analysis of covariance (ANCOVA) to study the differences in different blood biomarkers among the ID groups using age as a covariate. Only those biomarkers which exhibited significant difference among the ID groups were reported along with their corresponding by *‘p’* values (Supplementary Tables 1–8).

Linear regression was performed with the biological parameters as dependent variables, and raw/observed IQ scores along with age as independent variables. For each biochemical parameter, a separate regression analysis was performed. Furthermore, we have performed multiple testing adjustments using Bonferroni correction (i.e., ‘*p’* values < 0.05/3 were considered as significant after performing ANCOVA or linear regression) for all the analyses (Supplementary Tables 1–8).

Association between different pair-wise combinations of parameters was evaluated using Pearson’s (for parametric biomarkers) or Spearman’s (for non-parametric biomarkers) product moment correlation (r) method. Values of product moment r ranging from 0.8–1.0 were considered as strong association among the parameters (Supplementary Tables 10–16). Densitometric analyses of the western blots, surface plot of RBC and intensity curve of immunofluorescence images were obtained using ImageJ and OriginLab 8.0 softwares.

## Supplementary information

Supplementary material
