# Supplementary material for: An association study of severity of intellectual disability with peripheral biomarkers of disabled children in a rehabilitation home, Kolkata, India
Source: Sci Rep. 2019 Sep 20;9:13652. doi: 10.1038/s41598-019-49728-3 (PMC6754507; doi:10.1038/s41598-019-49728-3)
Supplement: Supplementary file 1 — Supplementary material [file 41598_2019_49728_MOESM1_ESM.pdf]

Supplementary materials

Title Page

Title: An association study of severity of intellectual disability with peripheral biomarkers of disabled children in a rehabilitation home, Kolkata, India**.**

Authors

Aaveri Sengupta[1], Ujjal Das[1], Krishnendu Manna[1], Sushobhan Biswas[1], Siddhartha Datta[1], Amitava Khan[1], Tuhin Bhattacharya[1], Samrat Saha[1], Tapashi Mitra[2], Swapan Mukherjee[2], Anup K Sadhu[3], Suhrita Paul[4], Saurabh Ghosh[5], Rakhi Dey Sharma[6], Sanjit Dey[1*]

Author Affiliations

[1]Department of Physiology, University of Calcutta, 92, A.P.C Road, Kolkata-700009, West Bengal, India.

[2]Institute of Child Health, 11, Dr. Biresh Guha Street, Kolkata 700017, West Bengal, India.

[3]EKO CT and MRI Scan Centre, Medical College and Hospitals campus, 88, College Street, Kolkata 700073, West Bengal, India.

[4]Murshidabad Medical College and Hospital, 73, Station Road, Raninagar, Gora Bazar, Berhampore, West Bengal 742101, India.

[5]Human Genetics Unit, Indian Statistical Institute, 203 B.T. Road, Kolkata 700108, West Bengal, India.

[6]Department of Food and Nutrition, Barrackpore Rastraguru Surendranath College, 85, Middle Road, Kolkata 700 120, West Bengal, India.

Name and address of the corresponding author

Professor Sanjit Dey (Ph.D.), Department of Physiology, DST-PURSE & UGC-CPEPA supported Department, Centre for Research in Nanoscience & Nanotechnology (CRNN), University of Calcutta, 92, A.P.C Road, Kolkata 700009, West Bengal, India. E-mail: sanjitdey@gmail.com; Fax: +91-33-23519755; Tel: +91-9830211512.

Author contact details

1. Aaveri Sengupta, M.Sc.

Contact number: +91-9748205293.

E-mail: aaveri3009@gmail.com

2. Ujjal Das, M.Sc.

Contact number: +91-7980261098.

E-mail: ujjald78@gmail.com

3. Krishnendu Manna, Ph.D.

Contact number: +91-9830958006.

E-mail: mannakrishnendu.rocks@gmail.com

4. Sushobhan Biswas, M.Sc.

Contact number: +91-9804190289.

sushobhanbiswas03@gmail.com

5. Siddhartha Datta, Ph.D.

Contact number: +91-9836606844.

E-mail: sidhu_85chn@yahoo.co.in

6. Amitava Khan, Ph.D.

Contact number: +91-9051150127.

E-mail: amitava84@gmail.com

7. Tuhin Bhattacharya, M.Sc.

Contact number: +91-7872352449.

E-mail: tuhin.007.tb@gmail.com

8. Samrat Saha, M.Sc.

Contact number: +91-9804943948.

E-mail: aryasaha30@gmail.com

9. Tapashi Mitra, Ph.D.

Contact number: +91-9830603745.

E-mail: tapashi.mitra27@gmail.com

10. Swapan Mukherjee, MD.

Contact number: +91-9831037716.

E-mail: docswap@hotmail.com

11. Anup K Sadhu, MD.

Contact number: 033 2212-3778.

E-mail: sadhujee1@gmail.com

12. Suhrita Paul, MD.

Contact number: +91-9830211512.

E-mail: drsuhritapaul@yahoo.co.in

13. Saurabh Ghosh, Ph.D.

Contact number: +91-9830780799.

E-mail: saughosh@gmail.com

14. Rakhi Dey Sharma, Ph.D.

Contact number: +91-9830166558.

E-mail: rakhidey70@gmail.com

Contents

**A. Supplementary Table. S1.**	**vii-viii**
**B. Supplementary Table. S2.**	**ix-x**
**C. Supplementary Table. S3.**	**xi-xii**
**D. Supplementary Table S4.**	**xiii-xiv**
**E. Supplementary Table S5.**	**xv**
**F. Supplementary Table S6.**	**xvi-xvii**
**G. Supplementary Table S7.**	**xviii**
**H. Supplementary Table S8.**	**xix-xx**
**I. Supplementary Table S9.**	**xxi-xxii**
**J. Supplementary Table. S10.**	**xxiii-xxiv**
**K. Supplementary Table. S11.**	**xxv**
**L. Supplementary Table. S12.**	**xxvi**
**M. Supplementary Table. S13.**	**xxvii-xxviii**
**N. Supplementary Table. S14.**	**xxix**
**O. Supplementary Table. S15.**	**xxx**

P. Supplementary Fig. S1 **xxxi**

Q. Images of raw bots **xxxii**

R. Details of protein ladder used **xxxiii**

Supplementary Results

Variable	Test performed (ANCOVA)		Pair-wise comparison between groups						Linear Regression on IQ scores (Age adjusted)
	p value (ID groups)	*p* value (age)	0,1		0,2		1,2		
			P **value (scores)**	***P*** **value (age)**	***P*** **value (scores)**	***P*** **value (age)**	***P*** **value (scores)**	***P*** **value (age)**	
**Haemoglobin (gm%)**	0.022*	0.579	0.466	0.161	**0.007****	0.693	0.030*	0.678	0.061
**Erythrocyte count (millions/cu.mm)**	0.252	0.202	---------	---------	--------	-------	--------	--------	0.094
**Leukocyte count (cu.mm)**	0.206	0.608	---------	----------	--------	-------	--------	--------	0.831
**HCT (%)**	**0.006****	0.871	0.300	0.957	0.156	0.951	**0.003****	0.957	0.042*
**MCV (fl)**	0.032*	0.606	0.548	0.956	0.419	0.502	**0.012****	0.549	0.042*
**MCH (pg)**	0.294	0.329	----------	---------	--------	-------	--------	--------	0.452
**MCHC (%)**	0.214	0.883	----------	--------	--------	-------	--------	--------	0.246
**PLT (lakhs/cu.mm)**	0.325	0.441	-----------	---------	--------	-------	--------	--------	0.964
**Neutrophil (%)**	**<0.001****	0.029	**0.001****	0.043*	0.210	0.084	**0.002****	0.043*	0.783
**Eosinophil (%)**	0.119	0.125	---------	---------	--------	-------	--------	--------	0.779
**Lymphocyte (%)**	**<0.001***	0.034	**<0.001****	0.097	0.214	0.125	0.056	**0.001****	0.620
**Monocyte (%)**	0.183	0.326	---------	---------	--------	-------	--------	-------	0.276

Supplementary Table S1. The status of haematological parameters of the three tested groups of subjects (N=45). The numbers (0) represents mild group, (1) moderate group and (2) severe group of ID subjects. As we found variations in the age range between the three ID groups, we carried out a one-way analysis of covariance (ANCOVA) to study the differences in different blood biomarkers among the ID groups using age as a covariate. Linear regression was performed with the haematological parameters as dependent variables, and raw/observed IQ scores along with age as independent variables. For each parameter, a separate regression analysis was performed. Furthermore, we have performed multiple testing adjustments using Bonferroni correction (i.e., '*p*' values <0.05/3 were considered as significant after performing ANCOVA or linear regression) for all the analyses. (*) sign represents significant '*p*' values found without correction for multiple testing and (**) represents significant '*p*' values observed after correction for multiple testing.

Variable	Test performed (ANCOVA)		Pair-wise comparison between groups						Linear Regression on IQ scores (Age adjusted)
	p value (ID groups)	*p* value (age)	0,1		0,2		1,2		
			P value (scores)	*P* value (age)	*P* value (scores)	*P* value (age)	*P* value (scores)	*P* value (age)	
**Glucose fasting (mg/dL)**	0.354	0.987	--------	--------	---------	-------	---------	----------	0.218
**Urea (mg/dL)**	**0.006****	0.137	0.027*	0.447	0.038	0.742	0.146	**0.003****	0.930
**Creatinine (mg/dL)**	0.069	0.890	--------	--------	---------	-------	--------	---------	0.162
**Calcium (mg/dL)**	0.502	0.397	--------	--------	---------	-------	--------	---------	0.642
**Phosphorus (mg/dL)**	0.133	0.237	---------	--------	---------	-------	--------	---------	0.030*
**Sodium (mEqv/L)**	0.775	0.424	---------	--------	---------	-------	--------	---------	0.796
**Potassium (mEqv/L)**	0.133	0.237	---------	--------	---------	-------	--------	---------	0.241
**Iron (µg/dL)**	0.517	0.632	---------	--------	---------	-------	--------	---------	0.550
**CRP (mg/L)**	0.584	0.155	----------	--------	----------	-------	--------	---------	0.922

Supplementary Table S2. The changes in clinical parameters of the three tested groups of subjects (N=45). The numbers (0) represents mild group, (1) moderate group and (2) severe group of ID subjects. As we found variations in the age range between the three ID groups, we carried out a one-way analysis of covariance (ANCOVA) to study the differences in different blood biomarkers among the ID groups using age as a covariate. Linear

regression was performed with these parameters as dependent variables, and raw/observed IQ scores along with age as independent variables. For each parameter, a separate regression analysis was performed. Furthermore, we have performed multiple testing adjustments using Bonferroni correction (i.e., '*p*' values <0.05/3 were considered as significant after performing ANCOVA, linear regression or correlation) for all the analyses. (*) sign represents significant '*p*' values found without correction for multiple testing and (**) represents significant '*p*' values observed after correction for multiple testing.

Variable	Test performed (ANCOVA)		Pair-wise comparison between groups						Linear Regression on IQ scores (Age adjusted)
	p value (ID groups)	*p* value (age)	0,1		0,2		1,2		
			P **value (scores)**	***P*** **value (age)**	***P*** **value (scores)**	***P*** **value (age)**	***P*** **value (scores)**	***P*** **value (age)**	
**Triglyceride (mg/dL)**	**<0.001****	**0.006****	**0.001****	0.671	**<0.001****	**0.006****	**<0.001****	**0.004****	**0.001****
**Total cholesterol (mg/dL)**	**<0.001****	0.223	**0.002****	0.754	0.441	0.442	**<0.001****	0.089	0.298
**LDL cholesterol (mg/dL)**	**<0.001****	0.789	**<0.001****	**0.011****	**<0.001****	0.448	0.061	**<0.001****	**0.001****
**HDL cholesterol (mg/dL)**	**<0.001****	0.145	0.179	0.977	**<0.001****	0.243	**<0.001****	0.023*	**0.001****
**VLDL cholesterol (mg/dL)**	**<0.001****	0.115	**0.008****	0.760	**<0.001****	0.051	**<0.001****	0.252	**0.001****

Supplementary Table S3. The changes in lipid profile of the three groups of ID subjects (N=45). The numbers (0) represents mild group, (1) moderate group and (2) severe group of ID subjects. As we found variations in the age range between the three ID groups, we carried out a one-way analysis of covariance (ANCOVA) to study the differences in different blood biomarkers among the ID groups using age as a covariate. Linear regression was performed with these parameters as dependent variables, and raw/observed IQ scores along with age as independent variables. For each parameter, a separate regression analysis was performed. Furthermore, we have performed multiple testing adjustments using Bonferroni correction (i.e., '*p*' values

<0.05/3 were considered as significant after performing ANCOVA, linear regression or correlation) for all the analyses. (*) sign represents significant '*p*' values found without correction for multiple testing and (**) represents significant '*p*' values observed after correction for multiple testing.

Variable	Test performed (ANCOVA)		Pair-wise comparison between groups						Linear Regression on IQ scores (Age adjusted)
	***p* value (ID groups)**	***p* value (age)**	**0,1**		**0,2**		**1,2**		
			***P* value (scores)**	***P* value (age)**	***P* value (scores)**	***P* value (age)**	***P* value (scores)**	***P* value (age)**	
**SGPT (IU/L)**	0.593	0.630	--------	--------	-------	------	-------	--------	0.957
**SGOT (IU/L)**	0.753	0.613	--------	---------	-------	-------	-------	--------	0.924
**Alkaline phosphatase (IU/L)**	0.353	0.589	-------	--------	-------	-------	-------	--------	0.366
**Bilirubin (mg/dL)**	0.283	0.110	--------	---------	-------	-------	--------	--------	0.097
**Total protein (g/dL)**	0.183	0.044	0.219	0.060	0.195	0.206	0.364	0.078	0.017*
**Albumin (g/dL)**	0.408	0.598	---------	--------	-------	-------	--------	--------	0.830
**Globulin (g/dL)**	0.101	0.012*	**0.004****	**0.001****	0.227	0.135	0.996	0.082	0.052
**A/G ratio**	0.206	0.019*	**<0.001****	**<0.001****	0.410	0.196	0.161	0.162	0.208

Supplementary Table S4. The alterations in parameters related to liver function in the three groups of ID subjects (N=45). The numbers (0) represents mild group, (1) moderate group and (2) severe group of ID subjects. As we found variations in the age range between the three ID groups, we carried out a one-way analysis of covariance (ANCOVA) to study the differences in various blood biomarkers among the ID groups using age as a covariate. Linear regression was performed with these parameters as dependent variables, and raw/observed IQ scores along with age as independent variables. For each parameter, a separate regression analysis was performed. Furthermore, we have performed multiple testing adjustments using Bonferroni correction (i.e.,

‘*p*’ values <0.05/3 were considered as significant after performing ANCOVA, linear regression or correlation) for all the analyses. (*) sign represents significant ‘*p*’ values found without correction for multiple testing and (**) represents significant ‘*p*’ values observed after correction for multiple testing.

Variable	Test performed (ANCOVA)		Pair-wise comparison between groups						Linear Regression on IQ scores (Age adjusted)
	p value (ID groups)	*p* value (age)	0,1		0,2		1,2		
			P value (scores)	*P* value (age)	*P* value (scores)	*P* value (age)	*P* value (scores)	*P* value (age)	
**Serum lactate (µg/dl/µg of protein)**	**<0.001****	0.230	**<0.001****	0.119	**<0.001****	0.115	**0.001****	0.144	**0.001****
**Serum LDH-A (Expression with respect to internal control GAPDH)**	**<0.001****	0.172	**0.001****	0.222	**0.001****	0.247	**0.001****	0.301	**0.005****

Supplementary Table S5. The alterations in metabolic parameters from serum in the three groups of ID subjects (N=45). The numbers (0) represents mild group, (1) moderate group and (2) severe group of ID subjects. As we found variations in the age range between the three ID groups, we carried out a one-way analysis of covariance (ANCOVA) to study the differences in various blood biomarkers among the ID groups using age as a covariate. Linear regression was performed with these parameters as dependent variables, and raw/observed IQ scores along with age as independent variables. For each parameter, a separate regression analysis was performed. Furthermore, we have performed multiple testing adjustments using Bonferroni correction (i.e., '*p*' values <0.05/3 were considered as significant after performing ANCOVA, linear regression or correlation) for all the analyses. (*) sign represents significant '*p*' values found without correction for multiple testing and (**) represents significant '*p*' values observed after correction for multiple testing.

Variable	Test performed (ANCOVA)		Pair-wise comparison between groups						Linear Regression on IQ scores (Age adjusted)
	p **value (ID groups)**	***p*** **value (age)**	**0,1**		**0,2**		**1,2**		
			P **value (scores)**	***P*** **value (age)**	***P*** **value (scores)**	***P*** **value (age)**	***P*** **value (scores)**	***P*** **value (age)**	
**Lipid peroxidation in PBMC (TBARS formation in nMoles/mg of protein)**	**<0.001****	0.036	**0.007****	0.725	0.686	0.037*	**<0.001****	0.020*	0.906
**Reduced GSH in PBMC (nMoles/mg of protein)**	**0.010****	0.220	0.046*	0.351	0.659	0.181	**0.008****	0.163	0.085
**SOD activity in PBMC (U/mg of protein)**	**<0.001****	0.329	**<0.001****	0.595	**<0.001****	0.358	0.018*	0.197	**0.001****
**Lipid peroxidation in RBC (TBARS formation in nMoles/mg of protein)**	**<0.001****	0.064	**0.004****	0.029*	**<0.001****	0.154	**0.002****	0.136	**0.001****
**Reduced GSH in RBC (nMoles/mg of protein)**	**<0.001****	0.603	**0.005****	0.882	**<0.001****	0.712	**<0.001****	0.603	**0.001****
**SOD activity in RBC (U/mg of protein)**	**<0.001****	0.223	**0.001****	0.378	**<0.001****	0.155	**<0.001****	0.506	**0.001****
**Serum ROS (% of change in fluorescence/µg of protein)**	**<0.001****	0.045*	**<0.001****	0.291	**<0.001****	**0.009****	**<0.001****	0.068	**0.001****
**Homocysteine (µmol/L)**	**<0.001****	0.065	**0.001****	0.556	**<0.001****	0.137	**0.006****	0.021*	**0.001****

Supplementary Table S6. The alterations in oxidative stress biomarkers from serum in the three groups of ID subjects (N=45). The numbers (0) represents mild group, (1) moderate group and (2) severe group of ID subjects. As we found variations in the age range between the three ID groups, we carried out a one-way analysis of covariance (ANCOVA) to study the differences in various blood biomarkers among the ID groups using age as a covariate. Linear regression was performed with these parameters as dependent variables, and raw/observed IQ scores along with age as independent variables. For each parameter, a separate regression analysis was performed. Furthermore, we have performed multiple testing adjustments using Bonferroni correction (i.e., '*p*' values <0.05/3 were considered as significant after performing ANCOVA, linear regression or correlation) for all the analyses. (*) sign represents significant '*p*' values found without correction for multiple testing and (**) represents significant '*p*' values observed after correction for multiple testing.

Variable	Test performed (ANCOVA)		Pair-wise comparison between groups						Linear Regression on IQ scores (Age adjusted)
	***p* value (ID groups)**	***p* value (age)**	**0,1**		**0,2**		**1,2**		
			***P* value (scores)**	***P* value (age)**	***P* value (scores)**	***P* value (age)**	***P* value (scores)**	***P* value (age)**	
**Glutamate (µg/ml/mg of protein)**	**<0.001****	0.047*	**0.001****	0.432	**<0.001****	0.094	**<0.001****	0.048*	**0.001****
**GABA (ng/ml/mg of protein)**	**<0.001****	0.335	**<0.001****	0.905	**<0.001****	0.539	0.411	0.145	0.030*
**Serotonin (ng/ml/mg of protein)**	**<0.001****	0.066	**<0.001****	0.880	0.644	0.048*	**<0.001****	0.091	0.115
**Dopamine (ng/ml/mg of protein)**	**<0.001****	0.094	**<0.001****	0.950	**<0.001****	0.088	**<0.001****	0.030*	0.046*

Supplementary Table S7. The alterations in serum neurotransmitter levels of three groups of ID subjects (N=45). The numbers (0) represents mild group, (1) moderate group and (2) severe group of ID subjects. As we found variations in the age range between the three ID groups, we carried out a one-way analysis of covariance (ANCOVA) to study the differences in various blood biomarkers among the ID groups using age as a covariate. Linear regression was performed with these parameters as dependent variables, and raw/observed IQ scores along with age as independent variables. For each parameter, a separate regression analysis was performed. Furthermore, we have performed multiple testing adjustments using Bonferroni correction (i.e., ‘*p*’ values <0.05/3 were considered as significant after performing ANCOVA, linear regression or correlation) for all the analyses. (*) sign represents significant ‘*p*’ values found without correction for multiple testing and (**) represents significant ‘*p*’ values observed after correction for multiple testing.

**Variable**	**Test performed (ANCOVA)**		**Pair-wise comparison between groups**						**Linear Regression on IQ scores (Age adjusted)**
	***p* value (ID groups)**	***p* value of (age)**	**0,1**		**0,2**		**1,2**		
			***P* value (scores)**	***P* value (age)**	***P* value (scores)**	***P* value (age)**	***P* value (scores)**	***P* value (age)**	
**Serum TNF-α (Expression with respect to internal control GAPDH)**	0.807	0.775	--------	-------	--------	-------	--------	-------	0.571
**Serum IL-6 (Expression with respect to internal control GAPDH)**	0.582	0.988	---------	-------	--------	-------	--------	-------	0.421
**Serum BDNF (Expression with respect to internal control GAPDH)**	0.354	0.544	--------	------	--------	-------	-------	-------	0.415
**RBC β actin (Expression with respect to internal control GAPDH)**	0.110	0.235	---------	-------	--------	-------	--------	-------	0.384

Supplementary Table S8. The expression patterns of serum pro-inflammatory proteins, neurotrophic factor and RBC membrane β actin of three groups of ID subjects (N=45). The numbers (0) represents mild group, (1) moderate group and (2) severe group of ID subjects. As we found variations in the age range between the three ID groups, we carried out a one-way analysis of covariance (ANCOVA) to study the differences in various blood biomarkers among the ID groups using age as a covariate. Linear regression was performed with these parameters as dependent variables, and raw/observed IQ scores along with age as independent variables. For each parameter, a separate regression analysis was performed. Furthermore, we have performed multiple testing adjustments using Bonferroni correction (i.e., '*p*' values <0.05/3 were considered as significant after performing ANCOVA, linear

regression or correlation) for all the analyses. (*) sign represents significant ‘*p*’ values found without correction for multiple testing and (**) represents significant ‘*p*’ values observed after correction for multiple testing.

	RBC count	MCH	HCT	MCV	Monocyte	Total WBC	Lymphocyte	Eosinophil	PLT	MCHC	Neutrophil	Hb
**RBC count**	---------											
**MCH**	0.2 (0.8)	-------										
**HCT**	0.0 (1.0)	0.4 (0.02)*	--------									
**MCV**	-0.2 (0.8)	0.58 **(0.001)****	0.7 **(0.001)****	--------								
**Monocyte**	0.0 (1.0)	0.2 (0.8)	0.4 (0.04)*	0.2 (0.8)	--------							
**Total WBC**	-0.1 (0.9)	0.1 (0.9)	0.2 (0.8)	0.2 (0.8)	0.4 (0.01)*	--------						
**Lymphocyte**	-0.1 (0.9)	0.1 (0.9)	0.05 (0.009)*	0.3 (0.09)	0.3 (0.09)	0.4 (0.01)*	---------					
**Eosinophil**	-0.3 (0.09)	-0.1 (0.9)	0.1 (0.9)	0.1 (0.9)	0.0 (1.0)	0.1 (0.9)	-0.3 (0.09)	---------				
**PLT**	0.0 (1.0)	-0.3 (0.1)	0.1 (0.9)	-0.2 (0.8)	0.3 (0.09)	-0.2 (0.8)	-0.3 (0.09)	0.1 (0.9)	-----			
**MCHC**	0.2 (0.8)	0.1 (0.9)	0.6 **(<0.001)****	-0.4 (0.01)*	-0.3 (0.09)	-0.1 (0.9)	-0.4 (0.02)*	-0.2 (0.8)	0.1 (0.9)	---------		
**Neutrophil**	0.0 (1.0)	-0.1 (0.9)	0.57 **(0.002)****	-0.3 (0.09)	-0.4 (0.01)*	-0.4 (0.01)*	-1.0 **(<0.001)****	0.1 (0.9)	0.2 (0.8)	0.4 (0.009)*	--------	
**Hb**	0.56 **(0.002)****	0.4 (0.03)*	0.5 (0.004)*	0.5 (0.004)*	0.3 (0.09)	0.1 (0.9)	0.3 (0.09)	0.0 (1.0)	0.2 (0.8)	-0.3 (0.1)	-0.3 (0.1)	---

Supplementary Table S9. The statistical evaluations of correlation for paired haematological parameters measured in the present study with experimental conditions of mild vs. moderate vs. severe groups. The values represent correlation coefficient (r) and significance level (*p*) between pair wise parameters. (+) sign in “r” represents positive correlation i.e., similar kind of changes (either increase or decrease) found in both parameters. (-) sign represents negative correlation i.e., one parameter increases vs. other parameter decreases or vice-versa. (*) sign represents significant correlation found without correction for multiple testing and (**) represents significant correlation observed after performing correction for multiple testing.

	Creatinine	Phosphorus	Potassium	Glucose	Urea	Sodium	Calcium	CRP	Iron
**Creatinine**	---------								
**Phosphorus**	0.5 (0.01)*	---------							
**Potassium**	0.2 (0.6)	-0.3 (0.08)	---------						
**Glucose**	-0.3 (0.08)	0.0 (1.0)	0.3 (0.08)	---------					
**Urea**	0.1 (0.9)	-0.1 (0.8)	-0.2 (0.5)	0.3 (0.08)	---------				
**Sodium**	-0.5 (0.02)*	-0.3 (0.08)	-0.2 (0.5)	-0.1 (0.8)	0.1 (0.8)	---------			
**Calcium**	-0.2 (0.5)	0.2 (0.6)	-0.1 (0.8)	-0.1 (0.8)	0.1 (0.8)	0.1 (0.8)	---------		
**CRP**	-0.4 (0.01)*	0.1 (0.9)	-0.2 (0.5)	0.0 (1.0)	0.4 (0.04)*	0.5 (0.05)*	0.6 (0.01)*	---------	
**Iron**	0.2 (0.6)	0.1 (0.9)	-0.2 (0.6)	-0.1 (0.8)	-0.1 (0.8)	-0.1 (0.8)	0.1 (0.9)	-0.2 (0.5)	---------

Supplementary Table S10. The statistical evaluations of correlation for paired clinical parameters measured in the present study with experimental conditions of mild vs. moderate vs. severe groups. The values represent correlation coefficient (r) and significance level (*p*) between pair wise parameters. (+) sign in "r" represents positive correlation i.e., similar kind of changes (either increase or decrease) found in both parameters. (-) sign represents negative correlation i.e., one parameter increases vs. other parameter decreases or vice-versa. (*) sign

represents significant correlation found without correction for multiple testing and (**) represents significant correlation observed after performing correction for multiple testing.

	**Bilirubin**	**SGPT**	**SGOT**	**ALP**	**Total protein**	**Albumin**	**Globulin**	**A/G-ratio**
**Bilirubin**	---------							
**SGPT**	0.3 (0.17)	---------						
**SGOT**	0.3 (0.18)	1.0 **(<0.001)****	---------					
**ALP**	0.2 (0.42)	0.0 (1.0)	0.0 (1.0)	---------				
**Total protein**	0.3 (0.17)	0.0 (1.0)	0.0 (1.0)	0.0 (1.0)	---------			
**Albumin**	0.6 (0.008)*	0.2 (0.39)	0.2 (0.35)	0.4 (0.15)	0.1 (0.8)	---------		
**Globulin**	0.0 (1.0)	-0.1 (0.83)	-0.1 (0.61)	-0.2 (0.52)	0.8 **(<0.001)****	-0.5 (0.03)*	---------	
**A/G-ratio**	0.2 (0.42)	0.1 (0.9)	0.1 (0.78)	0.2 (0.44)	-0.6 **(0.004)****	0.7 **(0.001)****	-0.9 **(<0.001)****	---------

Supplementary Table S11. The statistical evaluations of correlation for paired parameters of lipid profile measured in the present study with experimental conditions of mild vs. moderate vs. severe groups. The values represent correlation coefficient (r) and significance level (*p*) between pair wise parameters. (+) sign in "r" represents positive correlation i.e. similar kind of changes (either increase or decrease) found in both parameters. (-) sign represents negative correlation i.e. one parameter increases vs. other parameter decreases or vice-versa. (*) sign represents significant correlation found without correction for multiple testing and (**) represents significant correlation observed after performing correction for multiple testing.

	Bilirubin	SGPT	SGOT	ALP	Total protein	Albumin	Globulin	A/G-ratio
**Bilirubin**	---------							
**SGPT**	0.3 (0.17)	---------						
**SGOT**	0.3 (0.18)	1.0 **(<0.001)****	---------					
**ALP**	0.2 (0.42)	0.0 (1.0)	0.0 (1.0)	---------				
**Total protein**	0.3 (0.17)	0.0 (1.0)	0.0 (1.0)	0.0 (1.0)	---------			
**Albumin**	0.6 (0.008)*	0.2 (0.39)	0.2 (0.35)	0.4 (0.15)	0.1 (0.8)	---------		
**Globulin**	0.0 (1.0)	-0.1 (0.83)	-0.1 (0.61)	-0.2 (0.52)	0.8 **(<0.001)****	-0.5 (0.03)*	---------	
**A/G-ratio**	0.2 (0.42)	0.1 (0.9)	0.1 (0.78)	0.2 (0.44)	-0.6 **(0.004)****	0.7 **(0.001)****	-0.9 **(<0.001)****	---------

Supplementary Table S12. The statistical evaluations of correlation for paired parameters related to liver function were measured in the present study with experimental conditions of mild vs. moderate vs. severe groups. The values represent correlation coefficient (r) and significance level (*p*) between pair wise parameters. (+) sign in “r” represents positive correlation i.e., similar kind of changes (either increase or decrease) found in both parameters. (-) sign represents negative correlation i.e., one parameter increases vs. other parameter decreases or vice-versa. (*) sign represents significant correlation found without correction for multiple testing and (**) represents significant correlation observed after performing correction for multiple testing.

	**Hcy-Serum**	**TBARS-RBC**	**ROS-Serum**	**TBARS-PBMC**	**SOD-RBC**	**SOD-PBMC**	**GSH-RBC**	**GSH-PBMC**
**Hcy-Serum**	---------							
**TBARS-RBC**	0.7 **(<0.001)****	---------						
**ROS-Serum**	0.8 **(<0.001)****	0.9 **(<0.001)****	---------					
**TBARS-PBMC**	-0.1 (0.5)	-0.4 (0.09)	-0.3 (0.1)	---------				
**SOD-RBC**	-0.8 **(<0.001)****	-0.8 **(<0.001)****	-0.8 **(<0.001)****	0.0 (1.0)	---------			
**SOD-PBMC**	-0.8 **(<0.001)****	-0.6 **(<0.001)****	-0.7 **(<0.001)****	-0.2 (0.3)	0.8 **(<0.001)****	---------		
**GSH-RBC**	-0.8 **(<0.001)****	-0.6 **(<0.001)****	-0.8 **(<0.001)****	0.0 (1.0)	0.8 **(<0.001)****	0.9 **(<0.001)****	---------	
**GSH-PBMC**	0.2 (0.1)	0.6 **(<0.001)****	0.5 (0.008)*	-0.6 **(<0.001)****	-0.3 (0.1)	0.0 (1.0)	-0.3 (0.15)	---------

Supplementary Table S13. The statistical evaluations of correlation for paired parameters related to oxidative stress were measured in the present study with experimental conditions of mild vs. moderate vs. severe groups. The values represent correlation coefficient (r) and significance level (*p*) between pair wise parameters. (+) sign in "r" represents positive correlation i.e., similar kind of changes (either increase or decrease) found in both parameters. (-) sign represents negative correlation i.e., one parameter increases vs. other parameter decreases or vice-versa. (*) sign represents significant correlation found without correction for multiple testing and (**) represents significant correlation

observed after performing correction for multiple testing.

	Glutamate	GABA	Serotonin	Dopamine
**Glutamate**	---------			
**GABA**	-0.7 **(<0.001)****	---------		
**Serotonin**	0.6 **(0.001)****	0.0 (9.9)	---------	
**Dopamine**	-0.9 **(<0.001)****	0.8 **(<0.001)****	-0.3 (0.1)	---------

Supplementary Table S14. The statistical evaluations of correlation for paired parameters of serum neurotransmitters were measured in the present study with experimental conditions of mild vs. moderate vs. severe groups. The values represent correlation coefficient (r) and significance level (*p*) between pair wise parameters. (+) sign in "r" represents positive correlation i.e., similar kind of changes (either increase or decrease) found in both parameters. (-) sign represents negative correlation i.e., one parameter increases vs. other parameter decreases or vice-versa. (*) sign represents significant correlation found without correction for multiple testing and (**) represents significant correlation observed after performing correction for multiple testing.

	**Serum Lactate**
**Serum LDH-A expression**	0.93 **(<0.001)****

Supplementary Table S15. The statistical evaluations of correlation for paired serum metabolites were measured in the present study with experimental conditions of mild vs. moderate vs. severe groups. The values represent correlation coefficient (r) and significance level (*p*) between pair wise parameters. (+) sign in "r" represents positive correlation i.e., similar kind of changes (either increase or decrease) found in both parameters. (*) sign represents significant correlation found without correction for multiple testing and (**) represents significant correlation observed after performing correction for multiple testing.

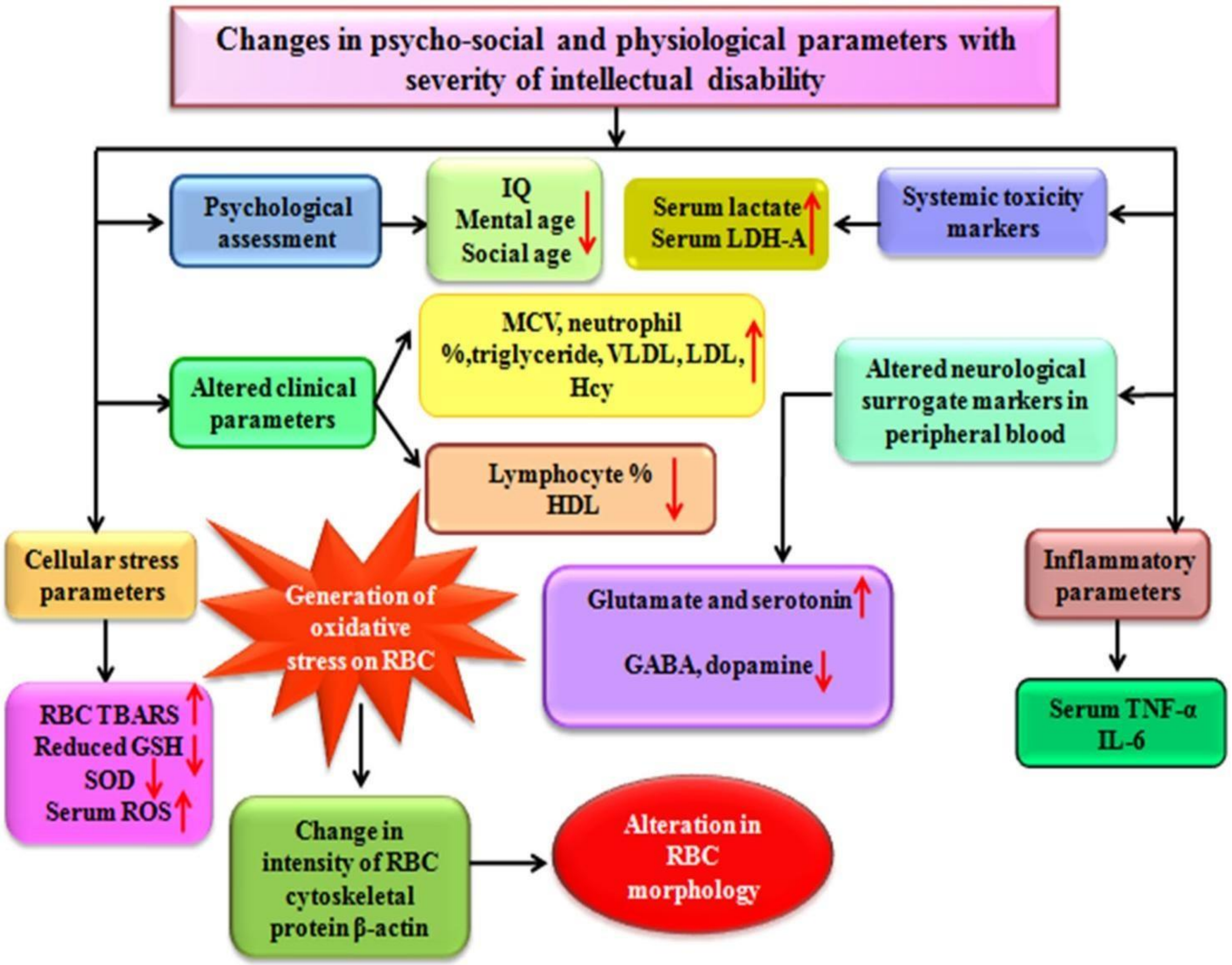

Supplementary Fig. S1. Schematic representation of the changes in psychological and physiological parameters with increasing degree of severity of intellectual disability.

Images of raw blots:

Fig. 7a. Serum BDNF

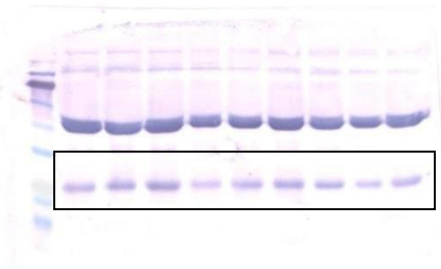

Fig. 7a. Serum TNF-α

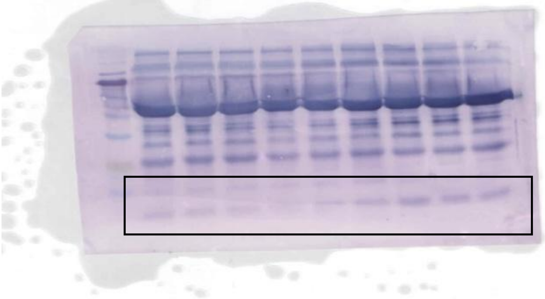

Fig. 6b. RBC β-actin

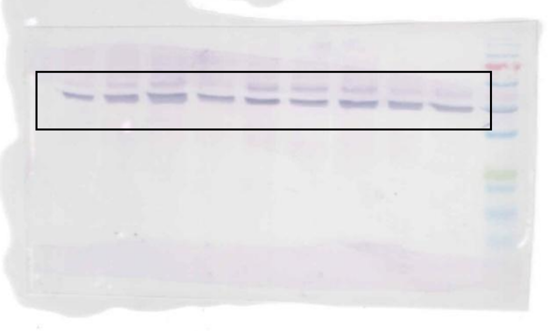

Fig. 6b. RBC GAPDH

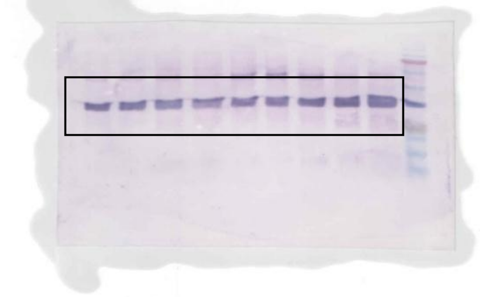

Fig. 7a. Serum IL-6

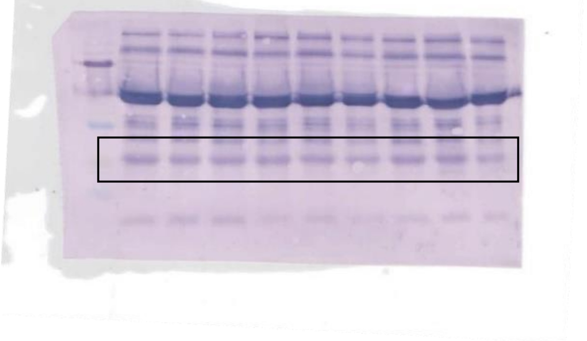

Fig. 7a. Serum GAPDH

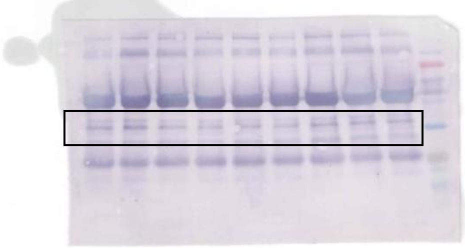

Details of the protein ladders used for immunoblots:

1. Precision plus protein standards, dual color, cat no. 161-0374, Biorad.

2. Prism ultra protein ladder (10-245 kDa) (ab116028).

3. Spectra Multicolor Broad range protein ladder from Thermo Fisher Scientific, cat no. 815-968-0747.
